# Supplementary material for: Cross-linking of the endolysosomal system reveals potential flotillin structures and cargo
Source: Nat Commun. 2022 Oct 20;13:6212. doi: 10.1038/s41467-022-33951-0 (PMC9584938; doi:10.1038/s41467-022-33951-0)
Supplement: Supplementary file 1 — Supplementary Information [file 41467_2022_33951_MOESM1_ESM.pdf]

Supplementary Information

Cross-linking of the Endolysosomal System Reveals Potential Flotillin Structures and Cargo

Jasjot Singh, Hadeer Elhabashy, Pathma Muthukottiappan, Markus Stepath, Martin Eisenacher, Oliver Kohlbacher, Volkmar Gieselmann, and Dominic Winter

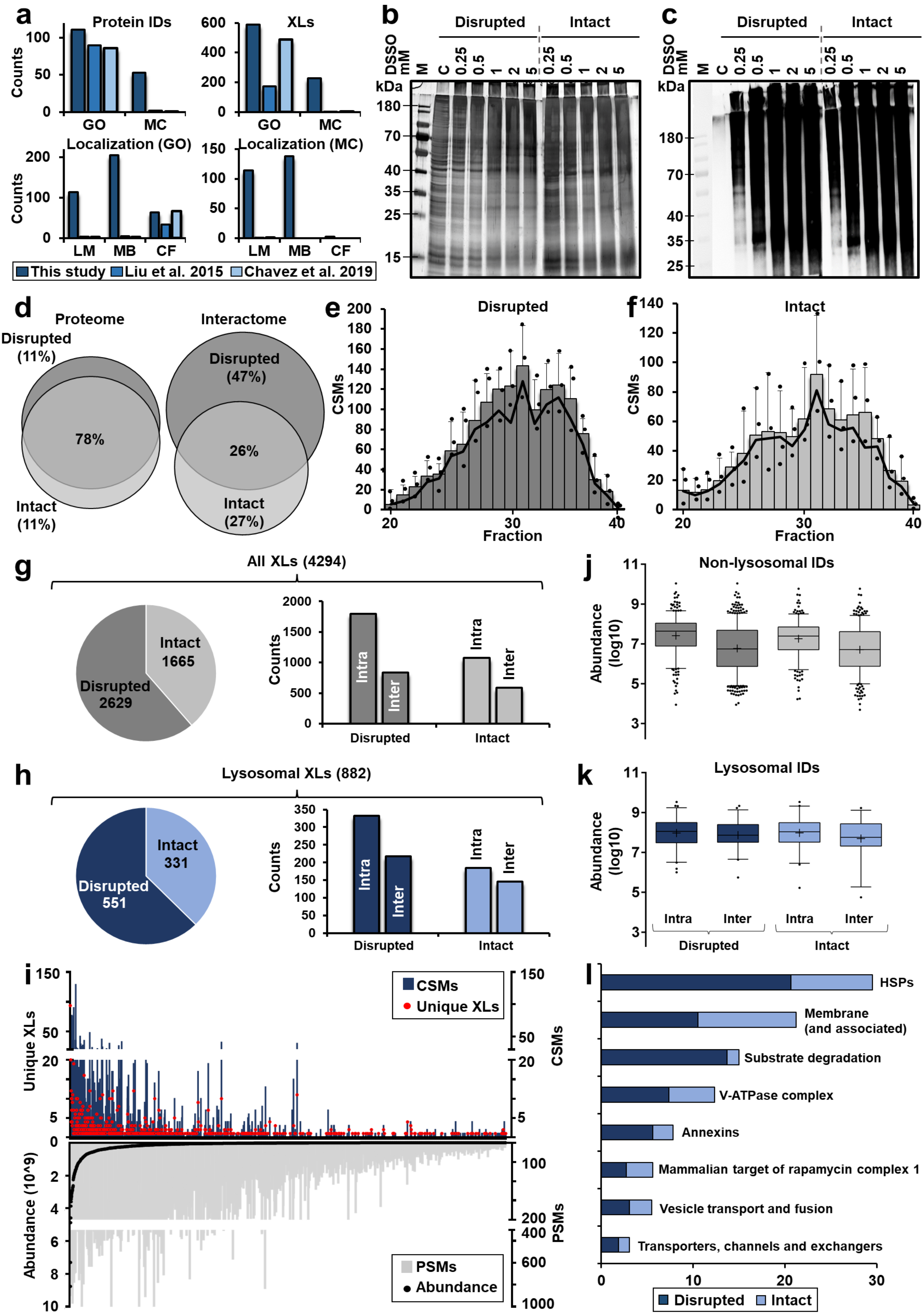
a
Protein IDs
XLs
Counts
GO
MC
Localization (GO)
Localization (MC)
LM
MB
CF
This study
Liu et al. 2015
Chavez et al. 2019
b
c
Disrupted
Intact
DSSO mM
M
C
0.25
0.5
1
2
5
kDa
180
70
40
35
25
15
d
Proteome
Interactome
Disrupted (11%)
78%
Intact (11%)
Disrupted (47%)
26%
Intact (27%)
e
Disrupted
f
Intact
CSMs
Fraction
g
All XLs (4294)
Intact 1665
Disrupted 2629
Intra
Inter
Counts
h
Lysosomal XLs (882)
Intact 331
Disrupted 551
j
Non-lysosomal IDs
Abundance (log10)
k
Lysosomal IDs
i
Unique XLs
CSMs
Unique XLs
Abundance (10^9)
PSMs
Abundance
l
HSPs
Membrane (and associated)
Substrate degradation
V-ATPase complex
Annexins
Mammalian target of rapamycin complex 1
Vesicle transport and fusion
Transporters, channels and exchangers
Disrupted
Intact

Supplementary Fig. 1: Cross-linking mass spectrometry analysis of HEK293 lysosome-enriched fractions.

a Identified cross-links for lysosomal and lysosome-related proteins. Proteins are categorized in two groups: GO: proteins which are related to lysosomes based on their categorization in gene ontology databases, also including proteins which are not important for lysosomal function; MC manually curated list of proteins which are localized in/at lysosomes [1] and known to play a role in lysosomal function. Numbers from published whole proteome cross-linking studies were extracted from the respective supplementary tables. Cross-links assigned to both GO and MC were further subcategorized based on the protein's location at the lysosome (lumen, membrane, and cytosolic face). **b** Silver-stained SDS-PAGE gel of lysosome-enriched fractions cross-linked with the indicated amounts of DSSO in a disrupted and intact state at a protein concentration of 1 µg/µL (n=2). For each sample, 2.5 µg of protein were loaded to the gel. Control samples were treated with DMSO. Based on the band pattern, the optimal concentration of DSSO was determined to be 0.25 mM (final concentration), as the application of higher amounts resulted in a shift of band patterns. Especially with respect to the lower molecular weight region, less bands were observed, while the higher molecular weight region (containing cross-linked proteins) increased in intensity, which is indicative for protein aggregates resulting from over-cross-linking. Shown are two separate SDS-PAGE analyses (performed under the same conditions) for samples cross-linked in the intact and the disrupted state. Individual gels are indicated by a dashed grey line. **c** Western blot analysis of samples described in (b) for the visualization of cross-linker-modified proteins using an in-house produced antibody which shows immunoreactivity against water-quenched DSSO[2] (n=2). For each sample, 1 µg of protein was loaded to the gel. Shown are two separate western blot analyses (performed under the same conditions) for samples cross-linked in the intact and the disrupted state. Individual gels are indicated by a dashed grey line. **d** Overlap in proteins, which are not categorized as lysosomal based on their GO term, identified in mass spectrometric analyses of lysosome-enriched fractions in the disrupted and the intact state. Proteome: proteins identified in analyses of non-cross-linked samples; Interactome: unique cross-links identified in analyses of cross-linked samples. **e, f** Distribution of identified CSMs in LC-MS/MS analyses of SCX-fractionated tryptic digests of lysosome-enriched fractions cross-linked in a disrupted (e) and intact (f) state. Since later eluting SCX fractions contain the majority of higher charged cross-linked tryptic peptides, only SCX fractions 20-40 were analyzed by LC-MS/MS. Data are presented as mean values +SD (n=3, biologically independent samples over 3 independent experiments). **g, h** Distribution of identified cross-links from the analysis of lysosome-enriched fractions cross-linked in the disrupted and the intact state. Shown are numbers identified in the disrupted/intact state, and how many cross-links were assigned as intra- or inter-links, for cross-links identified in the whole dataset (g) and such assigned to lysosomal proteins (h). **i** Correlation of cross-link identification and protein abundance for all non-lysosomal proteins. Proteins are sorted based on their mean iBAQ abundance[3] in the LC-MS/MS analysis of non-cross-linked samples. Mean iBAQ abundances as well as total numbers of unique cross-links, CSMs, and PSMs are shown. Values are based on the analysis of lysosome-enriched fractions in the intact and disrupted state (n=3 each). **j, k** Distribution of iBAQ abundances for all (j) and lysosomal (k) proteins for which intra- or inter-links were detected. Values are based on the LC-MS/MS analysis of non-cross-linked lysosome-enriched fractions in the disrupted and the intact state. Only proteins for which cross-links were identified in the respective state were considered. Data are presented as box-whisker plots (n=3, biologically independent samples over 3 independent experiments). Each box depicts the interquartile range (IQR, the range between the 25th and 75th percentile, 2.5% +/- whisker, median=line, mean=+). **i** Categorization of the 1,415 CSMs identified for 68 lysosomal proteins. Proteins were assigned to previously defined categories of lysosomal proteins [4].

IDs: identifications; LM: lumen; MB: membrane; CF: cytosolic face; GO: gene ontology; MC: manually curated; DSSO: disuccinimidyl sulfoxide; DMSO: dimethyl sulfoxide; C: control (no DSSO); M: protein marker; iBAQ: intensity based absolute quantification; SCX: strong cation exchange; XL: cross-link; PSMs: peptide spectral matches; CSMs: cross-link spectral matches; HSPs: heat shock proteins.

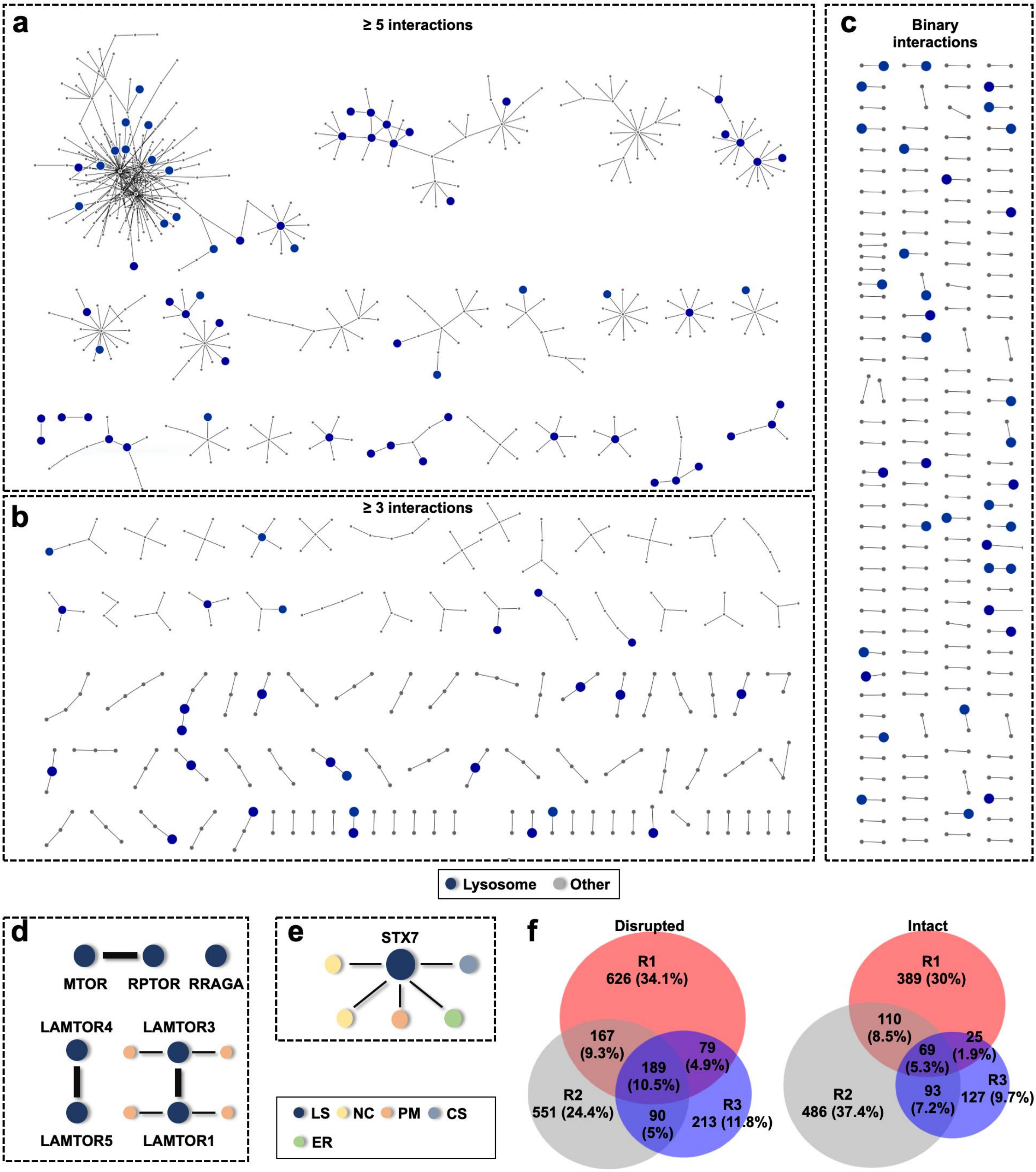
a
≥ 5 interactions
b
≥ 3 interactions
c
Binary interactions
Lysosome
Other
d
MTOR
RPTOR
RRAGA
LAMTOR4
LAMTOR3
LAMTOR5
LAMTOR1
e
STX7
LS
NC
PM
CS
ER
f
Disrupted
R1
626 (34.1%)
167
(9.3%)
79
(4.9%)
189
(10.5%)
R2
551 (24.4%)
90
(5%)
R3
213 (11.8%)
Intact
R1
389 (30%)
110
(8.5%)
25
(1.9%)
69
(5.3%)
R2
486 (37.4%)
93
(7.2%)
R3
127 (9.7%)

Supplementary Fig. 2: Analysis of protein-protein interactions in lysosome-enriched fractions. **a-c** Protein-protein interaction networks based on cross-links identified between two different proteins (inter-links, 1,023 in total). Lysosomal proteins are highlighted as large blue filled circles while non-lysosomal proteins are depicted as small grey dots. Interactions were extracted from the entire XL-LC-MS/MS dataset from disrupted and intact lysosomes. Based on the number of interactors involved in a particular network, data were grouped into different classes: a) ≥5 interactors, b) ≥3 interactors, c) binary interactions. The networks were generated using Cytoscape. **d** Protein-protein interaction networks for proteins related to mTORC1; subcellular localization of interactors is indicated by color. The networks were generated using Cytoscape. **e** Protein-protein interactions identified for Syntaxin 7 (STX7); subcellular localizations of interactors are indicated by color. The network was generated using Cytoscape. **f** Overlap of identified cross-links for the three individual biological replicates of cross-linked lysosome enriched fractions in a disrupted and intact state.
LS: lysosome; NC: nucleus; PM: plasma membrane; CS: cytoskeleton; ER: endoplasmic reticulum.

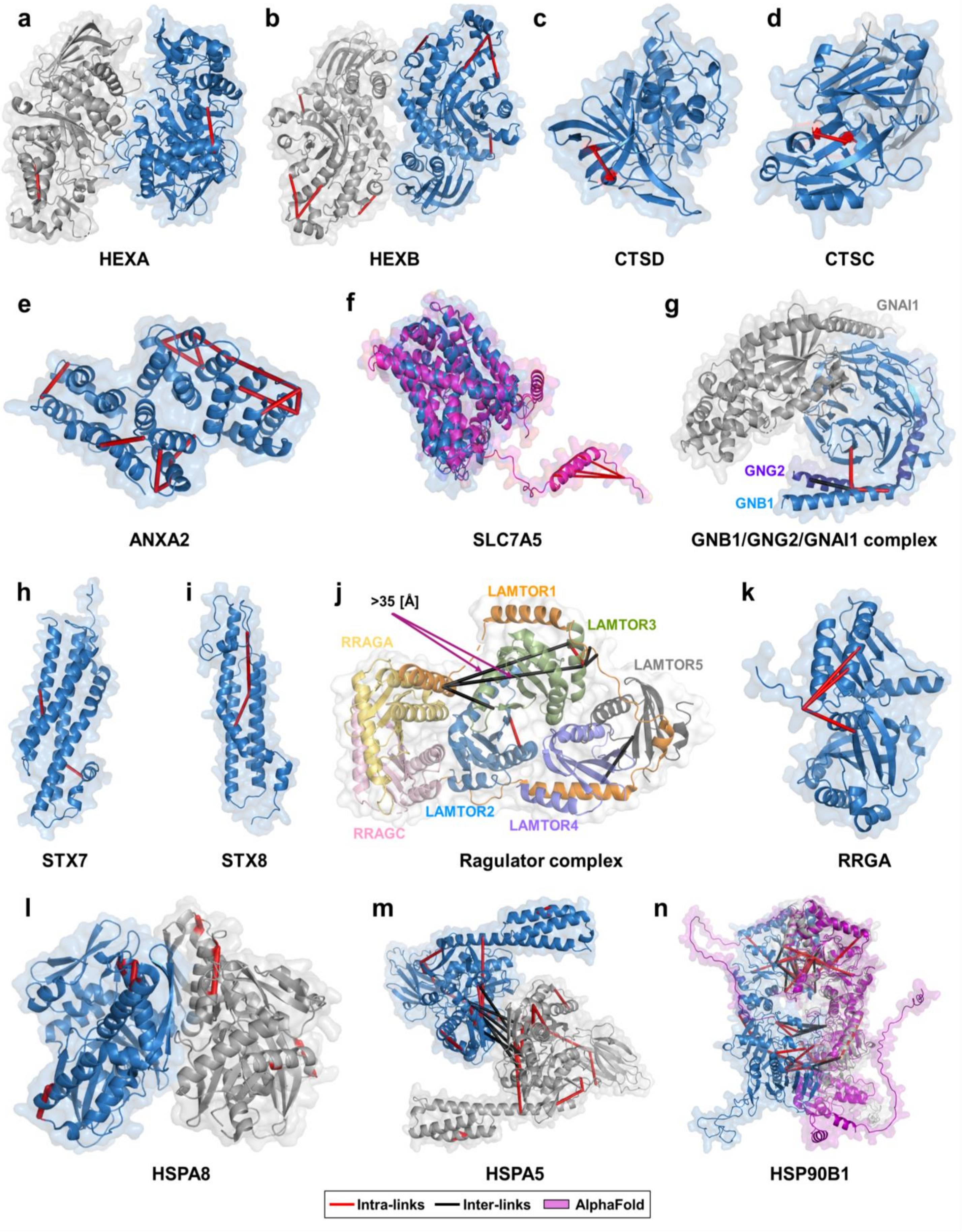
a
HEXA
b
HEXB
c
CTSD
d
CTSC
e
ANXA2
f
SLC7A5
g
GNAI1
GNG2
GNB1
GNB1/GNG2/GNAI1 complex
h
STX7
i
STX8
j
>35 [Å]
LAMTOR1
LAMTOR3
RRAGA
LAMTOR5
LAMTOR2
LAMTOR4
RRAGC
Ragulator complex
k
RRGA
l
HSPA8
m
HSPA5
n
HSP90B1
Intra-links
Inter-links
AlphaFold

Supplementary Fig. 3: Integration of cross-links into resolved structures and AlphaFold2 models of lysosomal and lysosome-associated proteins.
a Integration of cross-links into the RS of beta-hexosaminidase subunit alpha (HEXA). **b** Integration of cross-links into the RS of Beta-hexosaminidase subunit beta (HEXB). **c** Integration of cross-links into the RS of cathepsin D (CTSD). **d** Integration of cross-links into the RS of cathepsin C (CTSC).
e Integration of cross-links into the RS of annexin A2 (ANXA2). **f** Integration of cross-links into the AF [5] model of the large neutral amino acids transporter small subunit 1 (SLC7A5). **g** Integration of cross-links into the RS of the complex of GNB1, GNG2, and GNAI1. **h** Integration of cross-links into the homology model of syntaxin 7 (STX7). **i** Integration of cross-links into the homology model of syntaxin 8 (STX8). **j** Integration of cross-links into the RS of the Ragulator complex (LAMTOR1-5) in the presence of RRAGA and RRAGC. **k** Integration of cross-links into the AF model of Ras-related GTP-binding protein A (RRGA). **l** Integration of cross-links into the RS of heatshock protein 8 (HSPA8). **m** Integration of cross-links into the RS of heatshock protein 5 (HSPA5). **n** Integration of cross-links into the mixed model based on the RS and the AF model for heatshock protein 90B1 (HSP90B1). Magenta structures were extracted from AlphaFold while grey/blue structures are based on crystal or cryo-EM structures retrieved from PDB.
Inter-links (cross-links between two different proteins or two subunits of the same protein) are shown in black and intra-links (cross-links within the same protein) are shown in red. Overlength cross-links (>35 Å) are indicated.
RS: resolved structure; AF: AlphaFold2.

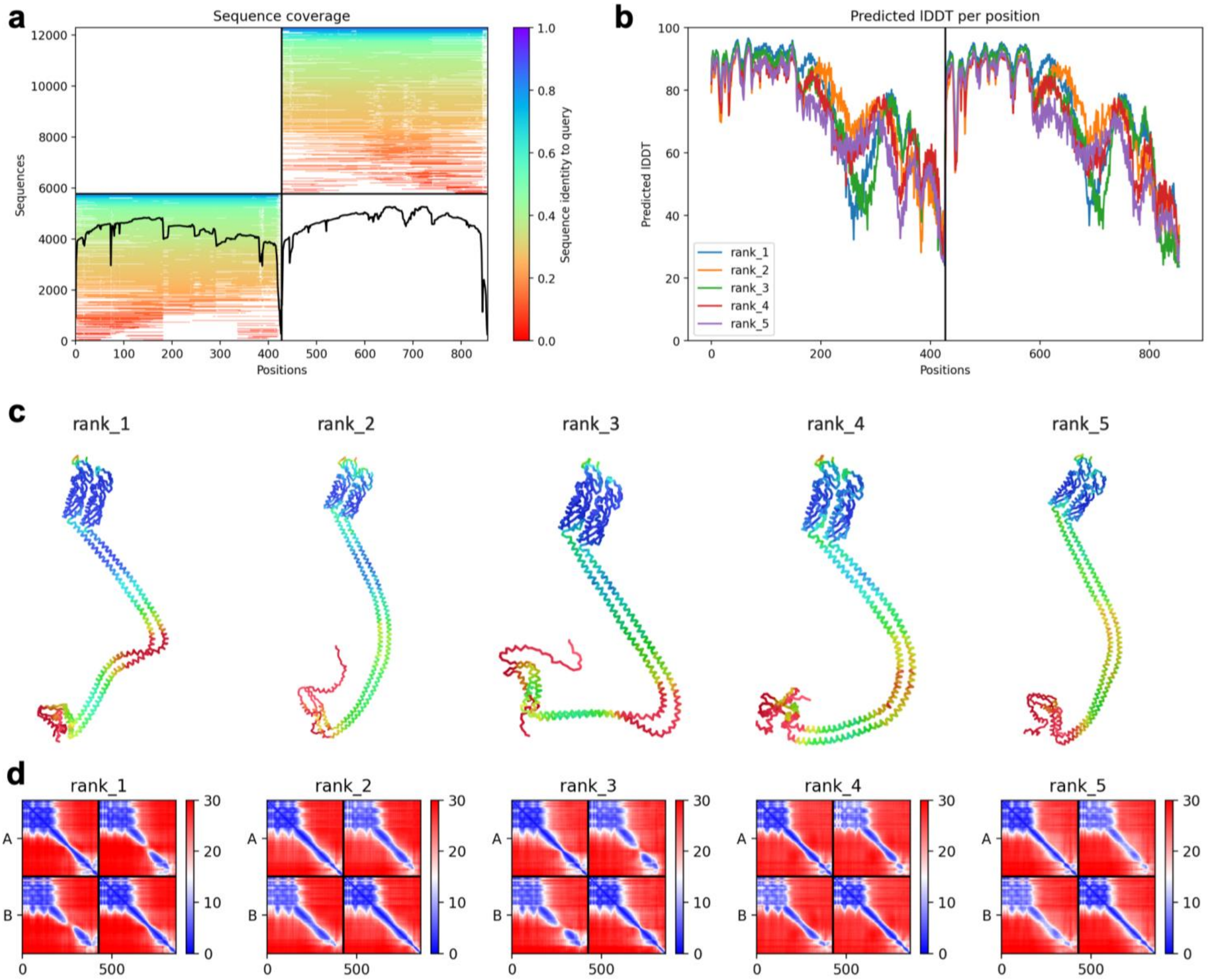

Supplementary Fig. 4: Quality assessment of different AlphaFold2-based ColabFold models of the FLOT1/FLOT2 heterodimeric complex.

a Visualization of depth and diversity of the multiple sequence alignment (MSA). **b** Predicted local distance difference test (plDDT) assessing local structural confidence of the different models. LDDT is a metric that evaluates local distance differences between all heavy atoms in a model, including validation of stereochemical plausibility. It ranges from 0 to 100, where 100 is the most confident. **c** Five highest scoring ColabFold models for the FLOT1-FLOT2 heterodimer. Color coding of individual structural features is based on their plDDTs per residue. **d** Inter-chain predicted aligned error (PAE), which aims to evaluate the position error at residue x, if the predicted and the true structures were aligned on residue y. It indicates the pairwise confidence of the respective prediction and ranks the models.

MSA: multiple sequence alignment; plDDT: predicted local distance difference test; PAE: predicted aligned error.

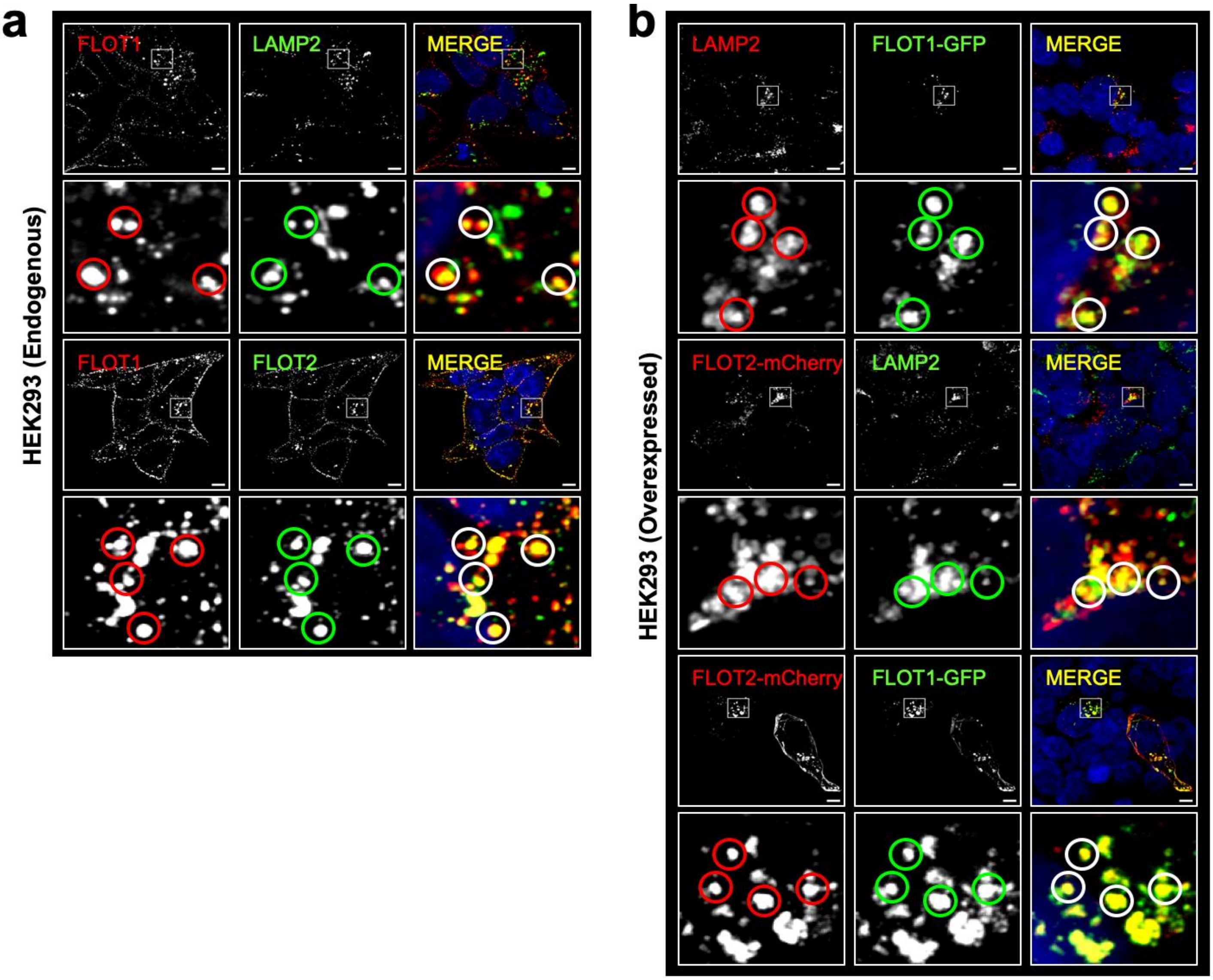

Supplementary Fig. 5: Investigation of FLOT1/FLOT2 distribution in HEK293 cells.
a Immunostaining of HEK293 cells for endogenous FLOT1, FLOT2, and the lysosomal marker LAMP2 (n=3). **b** Immunostaining of HEK293 cells transfected with FLOT1-GFP and/or FLOT2-mCherry in combination with staining for endogenous LAMP2. Images were acquired by confocal microscopy (n=2). The magnified region in the lower panel is indicated in the full-sized image. Scale bar = 5 µM.

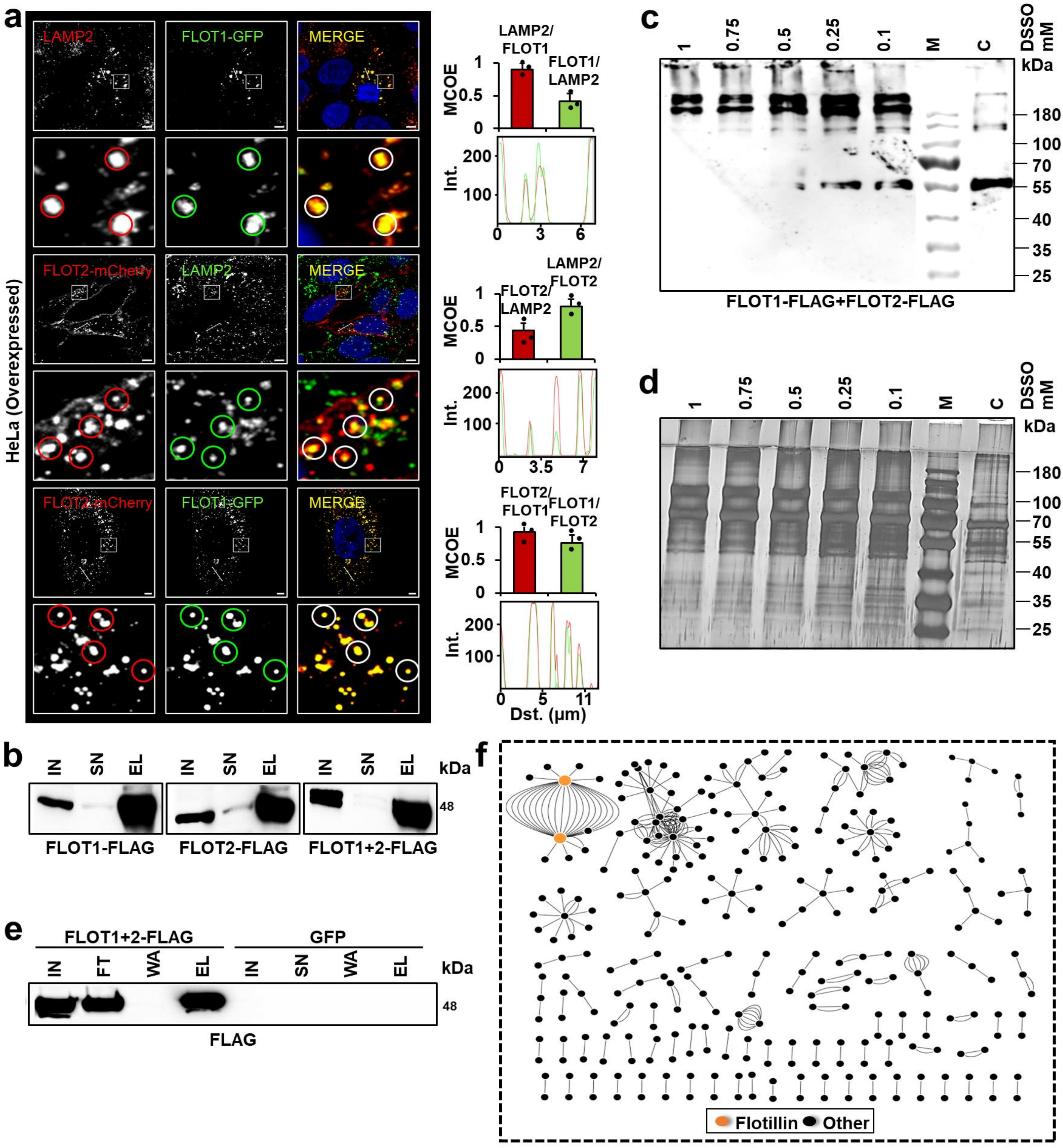
a
LAMP2
FLOT1-GFP
MERGE
FLOT2-mCherry
LAMP2
MERGE
FLOT2-mCherry
FLOT1-GFP
MERGE
HeLa (Overexpressed)
LAMP2/FLOT1
FLOT1/LAMP2
FLOT2/LAMP2
LAMP2/FLOT2
FLOT2/FLOT1
FLOT1/FLOT2
MCOE
Int.
Dst. (µm)
b
IN
SN
EL
kDa
48
FLOT1-FLAG
FLOT2-FLAG
FLOT1+2-FLAG
c
1
0.75
0.5
0.25
0.1
M
C
DSSO mM
kDa
180
100
70
55
40
35
25
FLOT1-FLAG+FLOT2-FLAG
d
e
FLOT1+2-FLAG
GFP
FT
WA
FLAG
f
Flotillin
Other

Supplementary Fig. 6: Investigation of higher order FLOT1/FLOT2 assemblies and XL-LC-MS/MS analysis of early endosomes.

a Immunostaining analysis of HeLa cells transiently transfected with FLOT1-GFP and/or FLOT2-mCherry constructs in combination with endogenous LAMP2. The magnified region in the lower panel is indicated in the full-size image. Mander's coefficients show the average degree of overlap of one protein population relative to another. Data are presented as mean values +SD (n=3, independent samples examined over one experiment). Profile plots indicate the degree of co-localization for individual vesicles. Scale bar = 5 µM. **b** Western blot analysis for verification of FLOT1-FLAG, FLOT2-FLAG, and FLOT1-FLAG+FLOT2-FLAG expression and enrichment by FLAG-IP (n=6). **c** Western blot analysis for determination of the optimal DSSO concentration for the cross-linking of SPIONs-enriched early endosomes from FLOT1-FLAG+FLOT2-FLAG transfected HEK293 cells (n=2). Early endosome-enriched fractions were cross-linked with the indicated amounts of DSSO at a protein concentration of 1 µg/µL followed by SDS-PAGE and anti-FLAG western blot. For each sample, 5 µg of protein were loaded to the gel. The non-cross-linked control sample (c) was treated with DMSO. Efficient cross-linking of FLOT1/FLOT2 complexes was judged by detection of the anti-FLAG band pattern. These analyses revealed that application of > 0.25 mM DSSO resulted in a near-quantitative loss of the FLOT1/FLOT2 monomers (~55 kDa) with almost exclusive detection of higher molecular weight assemblies (>180 kDa) and increasing amounts of protein aggregates which did not enter the separation gel. **d** Silver staining-based determination of the optimal DSSO concentration for the cross-linking of SPIONs-enriched early endosomes from FLOT1-FLAG+FLOT2-FLAG transfected HEK293 cells (n=1). Samples were generated and cross-linked as described in (c) followed by SDS-PAGE and silver staining of the gel. For each sample, 2.5 µg were loaded. The optimal DSSO concentration was determined to be 0.25 mM (final concentration), as the application of higher DSSO amounts resulted in reduced intensities in the low molecular weight region, indicating possible over cross-linking which could result in protein aggregates. Therefore, in accordance to the western blot results (see c), 0.25 mM DSSO was chosen. **e** Verification of presence of FLOT1 and FLOT2 in SPIONs-enriched early endosomes from FLOT1-FLAG+FLOT2-FLAG overexpressing HEK293 cells prior to the XL-LC-MS/MS experiment (n=6). Loading amount: 10 % of each fraction. **f** Interaction networks based on cross-links identified in the XL-LC-MS/MS dataset for DSSO-treated early endosome-enriched fractions from FLOT1-FLAG+FLOT2-FLAG-overexpressing HEK293 cells. In total, 324 inter-links were identified. FLOT1 and FLOT2 are highlighted in orange. The network was generated using Cytoscape. DSSO: disuccinimidyl sulfoxide; DMSO: dimethyl sulfoxide; PNS: post nuclear supernatant; M: marker; IN: input; SN: supernatant; FT: flow through; WA: wash; EL: eluate; C: control (no DSSO); IP: immunoprecipitation; BN-PAGE: blue native polyacrylamide gel electrophoresis; GFP: green fluorescent protein.

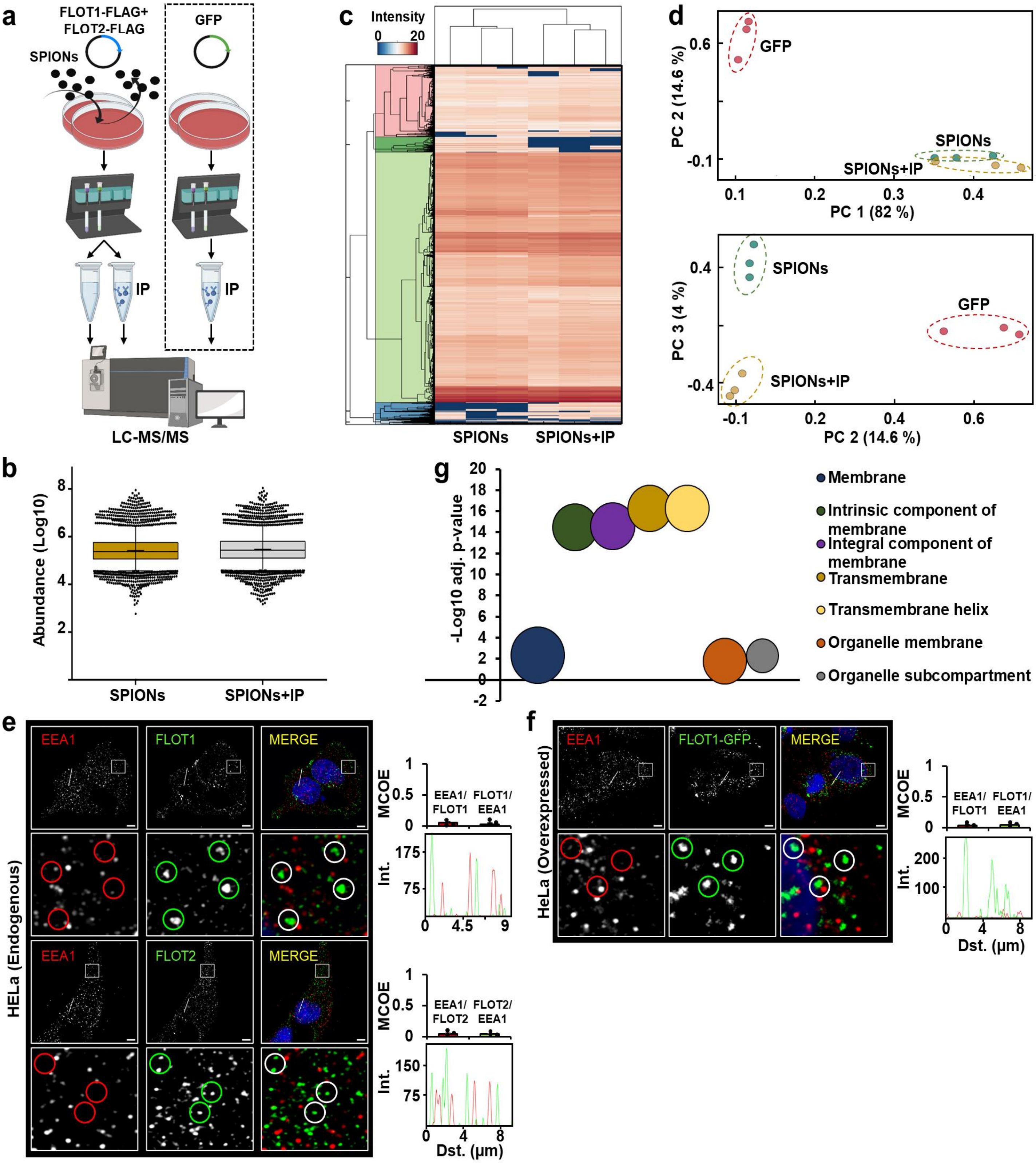
a
FLOT1-FLAG+
FLOT2-FLAG
SPIONs
GFP
IP
IP
LC-MS/MS
c
Intensity
0
20
SPIONs
SPIONs+IP
d
GFP
SPIONs
SPIONs+IP
PC 2 (14.6 %)
PC 1 (82 %)
SPIONs
GFP
SPIONs+IP
PC 3 (4 %)
PC 2 (14.6 %)
b
Abundance (Log10)
SPIONs
SPIONs+IP
g
-Log10 adj. p-value
Membrane
Intrinsic component of membrane
Integral component of membrane
Transmembrane
Transmembrane helix
Organelle membrane
Organelle subcompartment
e
HELa (Endogenous)
EEA1
FLOT1
MERGE
FLOT2
MCOE
EEA1/ FLOT1
FLOT1/ EEA1
EEA1/ FLOT2
FLOT2/ EEA1
Int.
Dst. (μm)
f
HeLa (Overexpressed)
EEA1
FLOT1-GFP
MERGE
MCOE
EEA1/ FLOT1
FLOT1/ EEA1
Int.
Dst. (μm)

Supplementary Fig. 7: Determination of flotillin vesicular cargo.

a Workflow for the enrichment of early endosomes by SPIONs and the subsequent enrichment of FLOT1-/FLOT2-positive early endosomal subpopulations by FLAG-IP. HEK293 cells were double-transfected with FLOT1-FLAG+FLOT2-FLAG, control cells with GFP. All samples were analyzed by LC-MS/MS using DIA. **b** Analysis of protein abundances in DIA datasets of SPIONs-enriched early endosomes and FLAG-IP-enriched FLOT1-/FLOT2-positive early endosomes. Data are presented as box-whisker plots (n=3, biologically independent samples over 3 independent experiments). Each box depicts the interquartile range (IQR, the range between the 25th and 75th percentile, 2.5% +/- whisker, median=line, average=+). **c** Unsupervised clustering of protein abundances (n=3, biologically independent samples over 3 independent experiments) for SPIONs-enriched early endosomes and FLAG-IP-enriched FLOT1-/FLOT2-positive early endosomes. Color coding correlates with the intensity of individual proteins. **d** PCA for all SPIONs-enriched early endosomes, FLAG-IP-enriched FLOT1-/FLOT2-positive early endosomes, and GFP-transfected control cells. Four individual principal components were used (PC1 and PC2, as well as PC3 and PC4). **e** Staining of endogenous levels of FLOT1, FLOT2, and EEA1 by confocal microscopy and investigation of co-localization. **f** Transient transfection of HeLa cells using FLOT1-GFP and co-staining with endogenous EEA1. **e/f** Images were acquired using confocal microscopy. The magnified region in the lower panel is indicated in the full-sized image. Mander's coefficients show the average degree of overlap of one protein population relative to another. Data are presented as mean values +SD (n=3, independent samples examined over one experiment). Profile plots indicate the degree of co-localization for individual vesicles. Scale bar = 5 µM. **g** GO enrichment analysis applying STRING for proteins which are overrepresented in FLOT1-/FLOT2-positive early endosomes. Results from the GO-category cellular component, as well as the UniProt keywords transmembrane and transmembrane helix are shown. Bubble size correlates with the number of proteins assigned to an individual category. Shown are p-values corrected for multiple testing within each category using the Benjamini–Hochberg procedure (cut off < 0.05) representing the significance of enrichment (n=3, biologically independent samples over 3 independent experiments).

SPIONs: superparamagnetic iron oxide nanoparticles; DIA: data independent acquisition; IP: immunoprecipitation; PCA: principal component analysis; adj.: adjusted; GO: gene ontology.

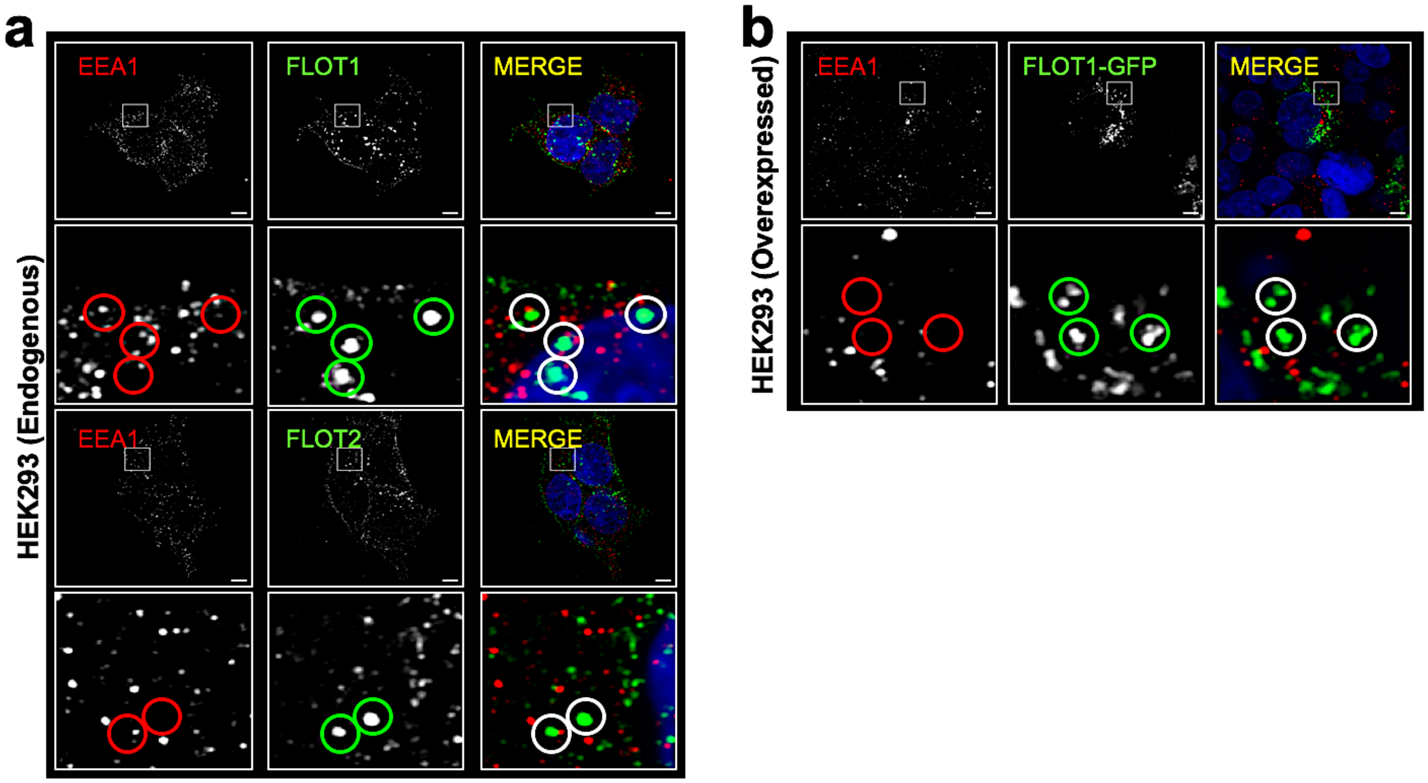

Supplementary Fig. 8: Investigation of EEA1 colocalization with FLOT1/FLOT2 in HEK293 cells. **a** Endogenous levels of FLOT1, FLOT2, and EEA1 were analyzed. The images were acquired using confocal microscopy (n=3). **b** HEK293 cells were transiently transfected using FLOT1-GFP and analyzed in combination with endogenous EEA1 staining. The images were acquired using confocal microscopy (n=2). The magnified images shown in the lower panel are indicated in the full-sized image. Scale bar = 5 µM.

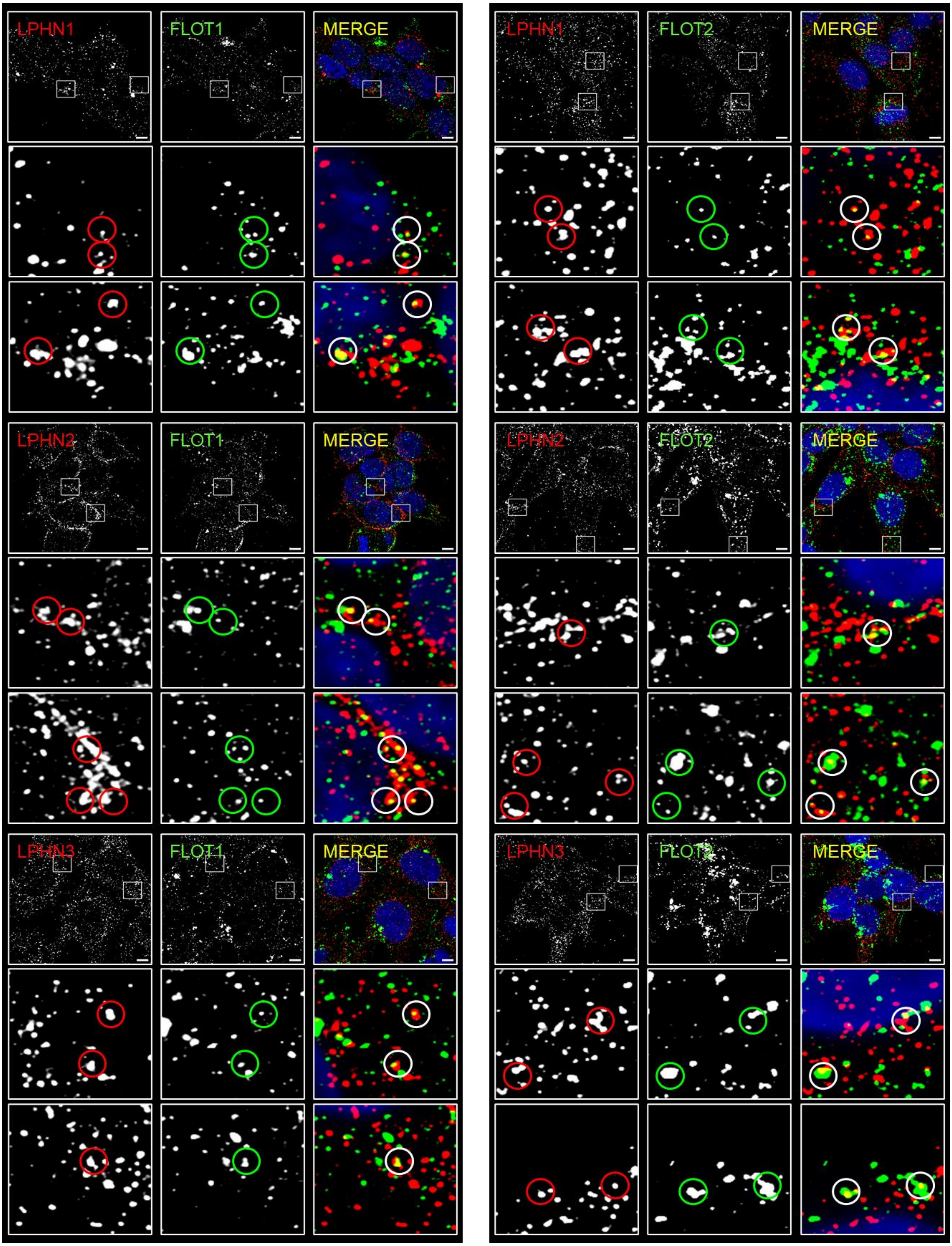
LPHN1
FLOT1
MERGE
LPHN1
FLOT2
MERGE
LPHN2
FLOT1
MERGE
LPHN2
FLOT2
MERGE
LPHN3
FLOT1
MERGE
LPHN3
FLOT2
MERGE

Supplementary Fig. 9: Latrophillins co-localize with FLOT1 and FLOT2 in punctate stainings. HEK293 cells were stained for FLOT1 and FLOT2 in combination with LPHN1, LPHN2, or LPHN3. Images were acquired using confocal microscopy (n=3). The magnified images shown in the lower panel are indicated in the full-sized image. Scale bar = 5 µM.

Supplementary References

1. Thelen, M., Winter, D., Braulke, T., Gieselmann, V. SILAC-Based Comparative Proteomic Analysis of Lysosomes from Mammalian Cells Using LC-MS/MS. *Methods Mol. Biol.* **1594**, 1–18 (2017).
2. Singh, J., Ponnaiyan, S., Gieselmann, V. & Winter, D. Generation of Antibodies Targeting Cleavable Cross-Linkers. *Anal. Chem.* **93**, 3762-3769 (2021).
3. Schwanhausser, B. *et al.* Global quantification of mammalian gene expression control. *Nature* **473**, 337-342 (2011).
4. Akter, F., Ponnaiyan, S., Kögler-Mohrbacher, Bi., Bleibaum, F., Damme, M., Renard, B.Y., Winter, D. Multi cell line analysis of lysosomal proteomes reveals unique features and novel lysosomal proteins. *bioRxiv* (2020).
5. Jumper, J. *et al.* Highly accurate protein structure prediction with AlphaFold. *Nature* **596**, 583-589 (2021).
