# Supplementary material for: Cross-linking of the endolysosomal system reveals potential flotillin structures and cargo
Source: Nat Commun. 2022 Oct 20;13:6212. doi: 10.1038/s41467-022-33951-0 (PMC9584938; doi:10.1038/s41467-022-33951-0)
Supplement: Supplementary file 3 — Description of Additional Supplementary Files [file 41467_2022_33951_MOESM3_ESM.docx]

**Description of Additional Supplementary Files**

**Supplementary Data 1: Lysosomal and lysosome-related proteins.**

**Table 1 (Lysosomal Proteins):** List proteins previously reported to be either located directly at the lysosome or to be related to the lysosome. Lysosome-localized proteins are based on a manually curated list (Thelen et al. 2017), lysosome-related proteins are based on a merged list from the public databases http://www.pantherdb.org/ and https://www.uniprot.org/. Only human proteins were considered. It is indicated in how many of the three databases each protein is listed.

**Supplementary Data 2: Proteins identified in DDA analyses of lysosome-enriched non-cross-linked samples.**

**Table 1 (All Proteins):** Includes all protein IDs identified in the DDA analysis of non-cross-linked lysosomes with LC-MSMS and data analysis with MaxQuant (exported at 1% FDR on peptide and protein level).

**Table 2 (All Proteins 3 Runs):** Protein IDs identified in at least three independent runs of the DDA analysis of non-cross-linked lysosomes with LC-MSMS and data analysis with MaxQuant (exported at 1% FDR on peptide and protein level).

**Table 3 (Lysosomal Proteins 3 Runs):** Lysosomal protein IDs identified in at least three independent runs of the DDA analysis of non-cross-linked lysosomes with LC-MSMS and data analysis with MaxQuant (exported at 1% FDR on peptide and protein level).

**Supplementary Data 3: Cross-links identified in XL-LC-MSMS analyses of lysosome-enriched fractions.**

**Table 1 (All XLs 5% FDR):** Cross links identified in the XL-LC-MS/MS analysis of disrupted and intact lysosome-enriched fractions based on the analysis with XlinkX and export at 5% FDR.

**Table 2 (All XLs 1% FDR):** Cross links identified in the XL-LC-MS/MS analysis of disrupted and intact lysosome-enriched fractions based on the analysis with XlinkX and export at 1% FDR.

**Supplementary Data 4: Cross-link distance constraints for selected proteins.**

**Table 1 (Intra-links):** Information for the structural validation of intra-links (cross-links within the same protein).

**Table 2 (Inter-links):** Information for the structural validation of inter-links (cross-links between two different proteins or subunits).

**Table 3 (Distance Constraint):** Distance constraint information for cross-links matched to proteins located at lysosomes and FLOT1/FLOT2-positive early endosomes including their Euclidean and topological distances between individual lysine residues.

**Supplementary Data 5: Proteins identified in DDA analyses of early endosome-enriched non-cross-linked samples.**

**Table 1 (All proteins):** Includes all protein IDs identified in the analysis of non-cross-linked early endosomes with LC-MSMS and data analysis with MaxQuant (exported at 1% FDR on peptide and protein level).

**Supplementary Data 6: Cross-links identified in XL-LC-MSMS analyses of early endosome-enriched samples.**

**Table 1 (All XLs 5% FDR):** Cross links identified in the XL-LC-MS/MS analysis of FLOT1/FLOT2-positive early endosome-enriched fractions based on the analysis with XlinkX and export at 5% FDR.

**Table 2 (All XLs 1% FDR):** Cross links identified in the XL-LC-MS/MS analysis of FLOT1/FLOT2-positive early endosome-enriched fractions based on the analysis with XlinkX and export at 1% FDR.

**Supplementary Data 7: Proteins and their DIA abundances for fractions obtained from the enrichment of FLOT1/FLOT2 positive early endosomes.**

**Table 1 (Non-Norm Abundance):** Proteins identified and quantified in all datasets based on the analysis of DIA files by Spectronaut and export with 1% FDR at peptide and protein level. Non-normalized DIA abundances are included.

**Table 2 (Norm Abundance):** Proteins identified and quantified in all datasets based on the analysis of DIA files by Spectronaut and export with 1% FDR at peptide and protein level. Normalized DIA abundances are included as well as fold change values as well significance levels (based on T-test).

**Table 3 (FLOT Cargo Candidates):** Proteins identified in 3/3 replicates for the SPIONs+IP samples and neither identified in the GFP or the SPIONs samples as well as all proteins significantly up-regulated in SPIONs+IP samples relative to SPIONs samples.

**Table 4 (FLOT Depleted Proteins):** Proteins significantly downregulated in 3/3 replicates for the SPIONs+IP sample (Fold Change >1.5)

**Table5 (Candidates GO Frequency):** Frequency of assignment of distinct GO-categories for all FLOT cargo candidates (Table 3).

**Table6 (Functional Clustering STRING):** Functional and physical protein protein interaction clusterings based on the STRING.

**Table7 (GO Enrichment Analysis):** Enrichment analysis of GO-categories

**Supplementary Data 8: Docking input/output parameters for PPT1 and all ranked PDB models.**

**Supplementary Data 9: Docking input/output parameters for FLOT1/FLOT2 and all ranked PDB models.**
