# Supplementary figures and images for: Cross-linking of the endolysosomal system reveals potential flotillin structures and cargo
Source: Nat Commun. 2022 Oct 20;13:6212. doi: 10.1038/s41467-022-33951-0 (PMC9584938; doi:10.1038/s41467-022-33951-0)

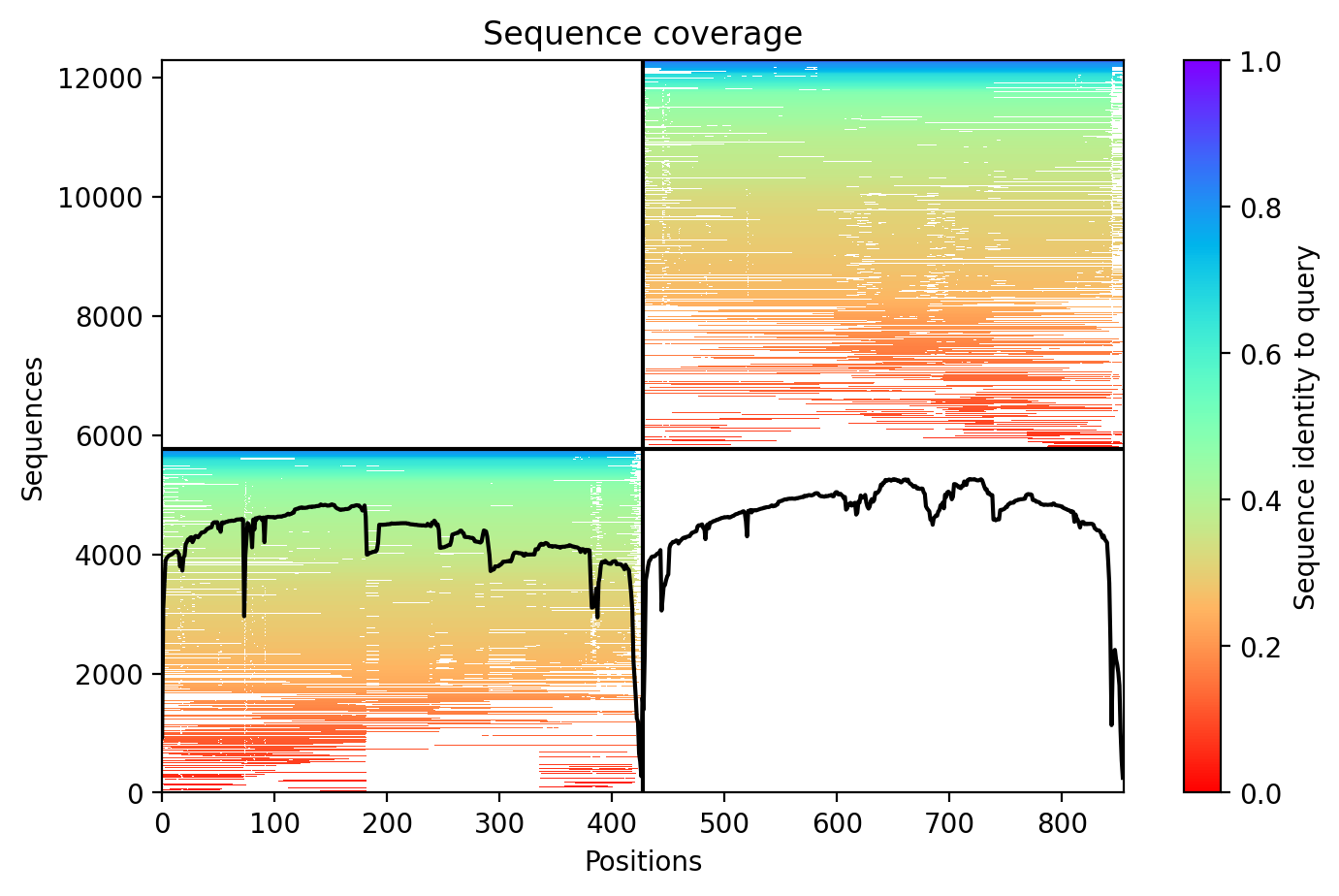

Supplement: Supplementary file 12 — Dataset 9 [file 41467_2022_33951_MOESM12_ESM.zip › FLOT1_FLOT2_dimer/msa_coverage.png]

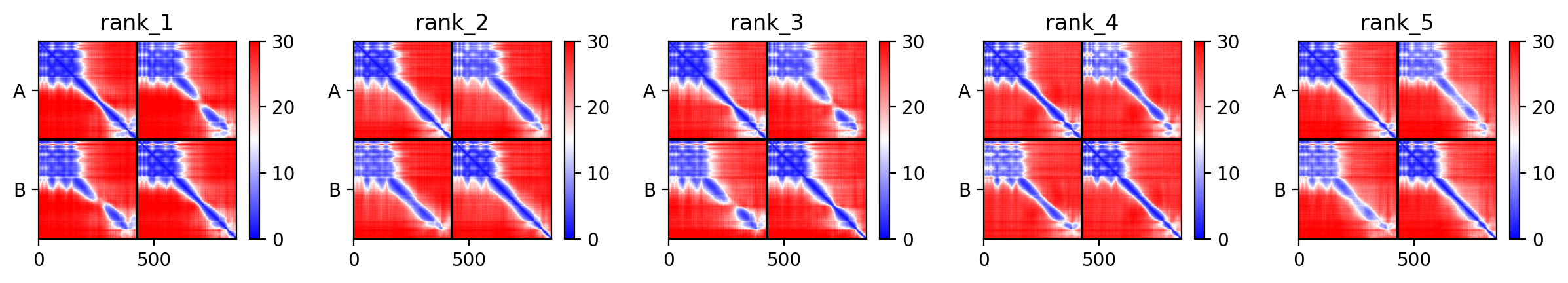

Supplement: Supplementary file 12 — Dataset 9 [file 41467_2022_33951_MOESM12_ESM.zip › FLOT1_FLOT2_dimer/predicted_alignment_error.png]

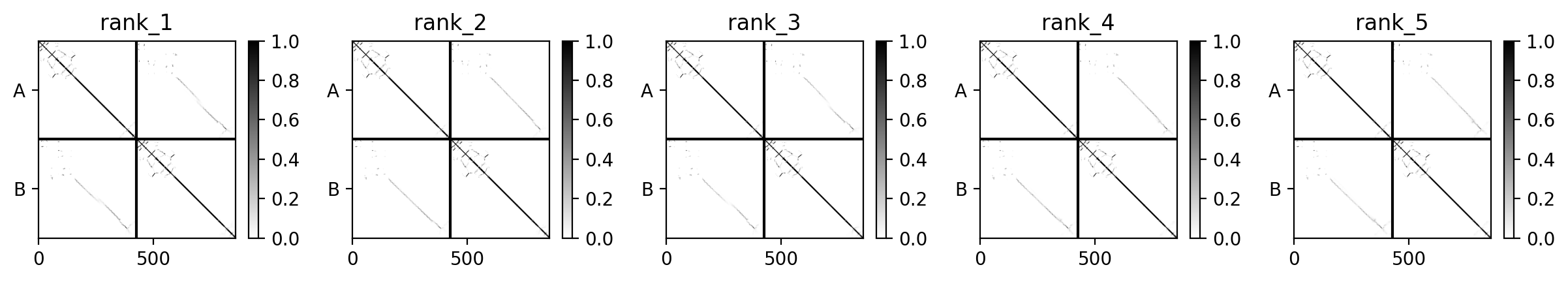

Supplement: Supplementary file 12 — Dataset 9 [file 41467_2022_33951_MOESM12_ESM.zip › FLOT1_FLOT2_dimer/predicted_contacts.png]

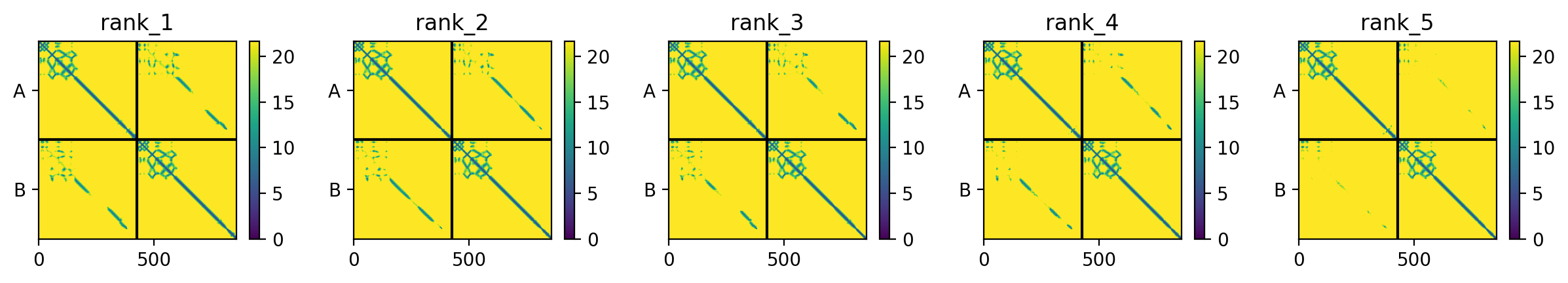

Supplement: Supplementary file 12 — Dataset 9 [file 41467_2022_33951_MOESM12_ESM.zip › FLOT1_FLOT2_dimer/predicted_distogram.png]

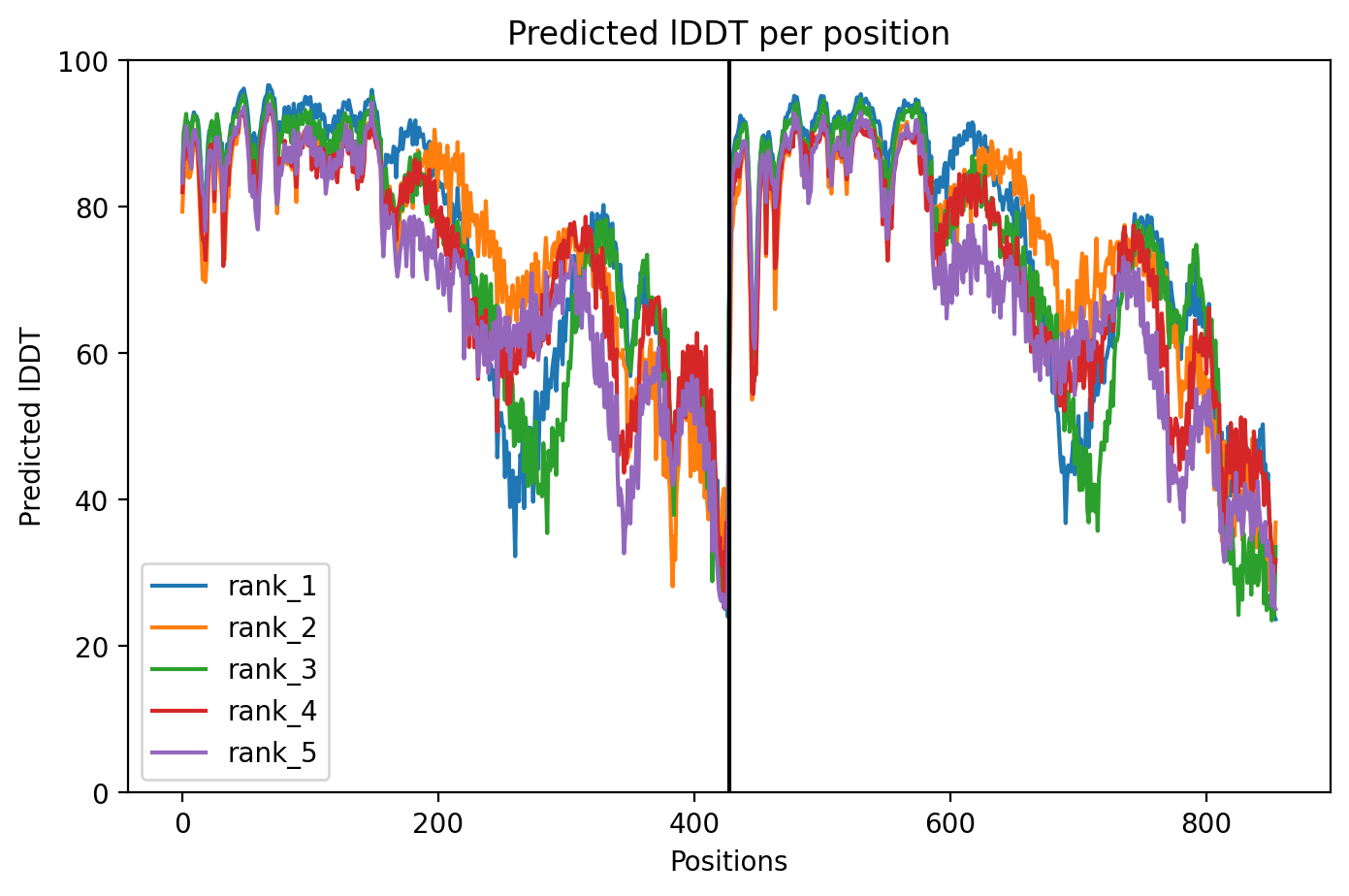

Supplement: Supplementary file 12 — Dataset 9 [file 41467_2022_33951_MOESM12_ESM.zip › FLOT1_FLOT2_dimer/predicted_LDDT.png]

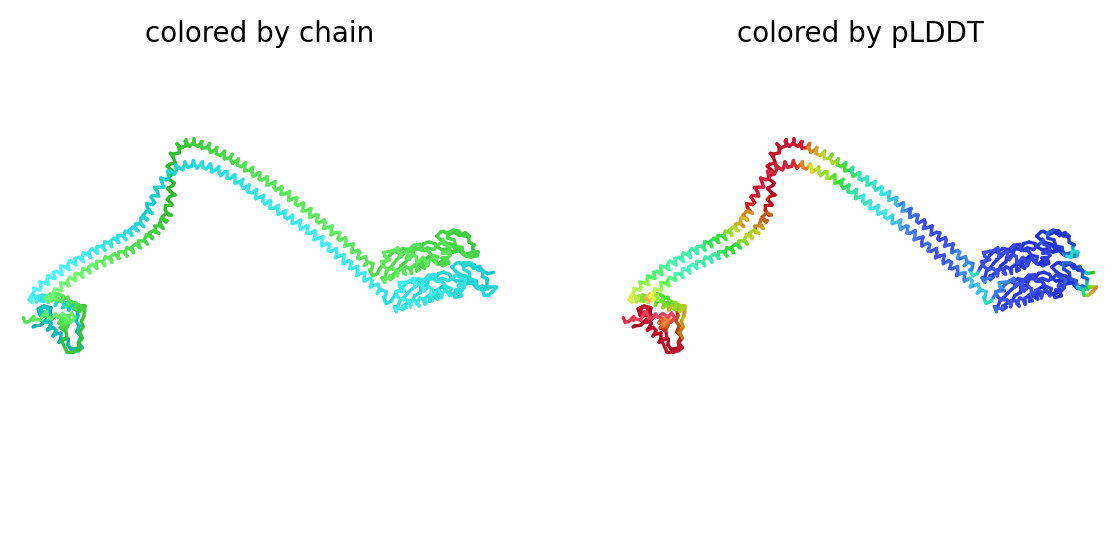

Supplement: Supplementary file 12 — Dataset 9 [file 41467_2022_33951_MOESM12_ESM.zip › FLOT1_FLOT2_dimer/rank_1_model_3_ptm_seed_0.png]

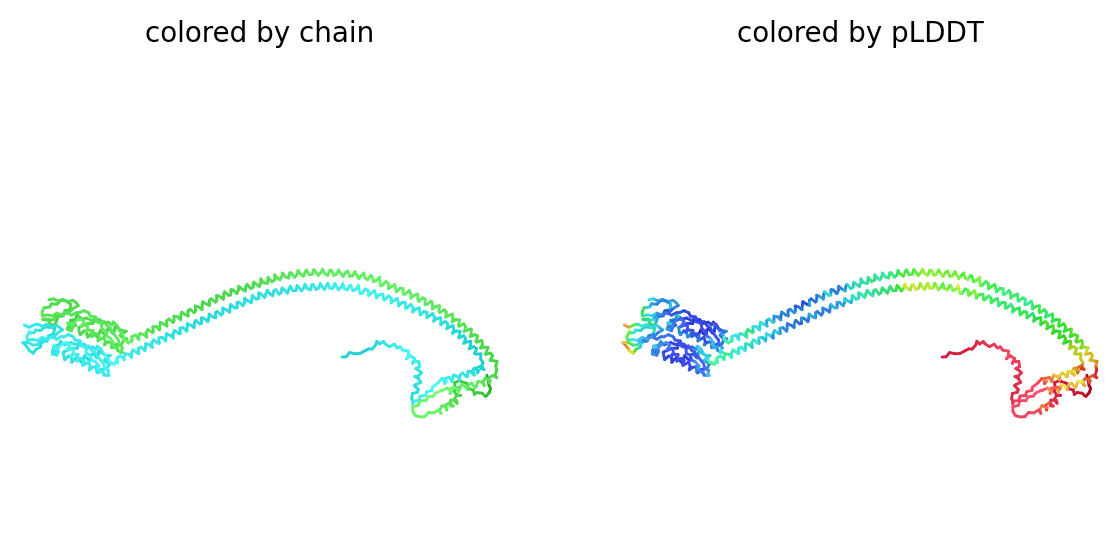

Supplement: Supplementary file 12 — Dataset 9 [file 41467_2022_33951_MOESM12_ESM.zip › FLOT1_FLOT2_dimer/rank_2_model_2_ptm_seed_0.png]

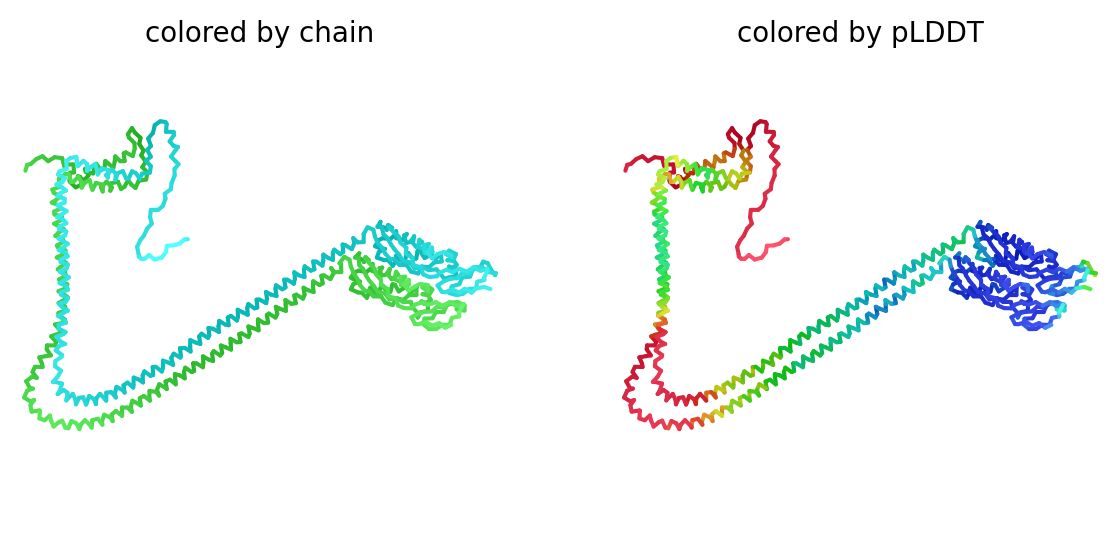

Supplement: Supplementary file 12 — Dataset 9 [file 41467_2022_33951_MOESM12_ESM.zip › FLOT1_FLOT2_dimer/rank_3_model_4_ptm_seed_0.png]

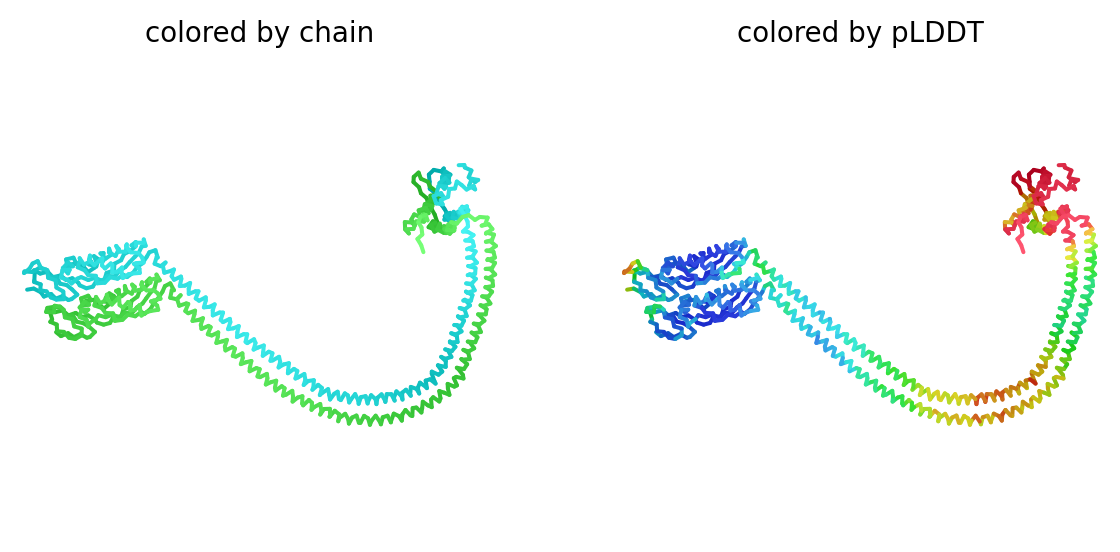

Supplement: Supplementary file 12 — Dataset 9 [file 41467_2022_33951_MOESM12_ESM.zip › FLOT1_FLOT2_dimer/rank_4_model_1_ptm_seed_0.png]

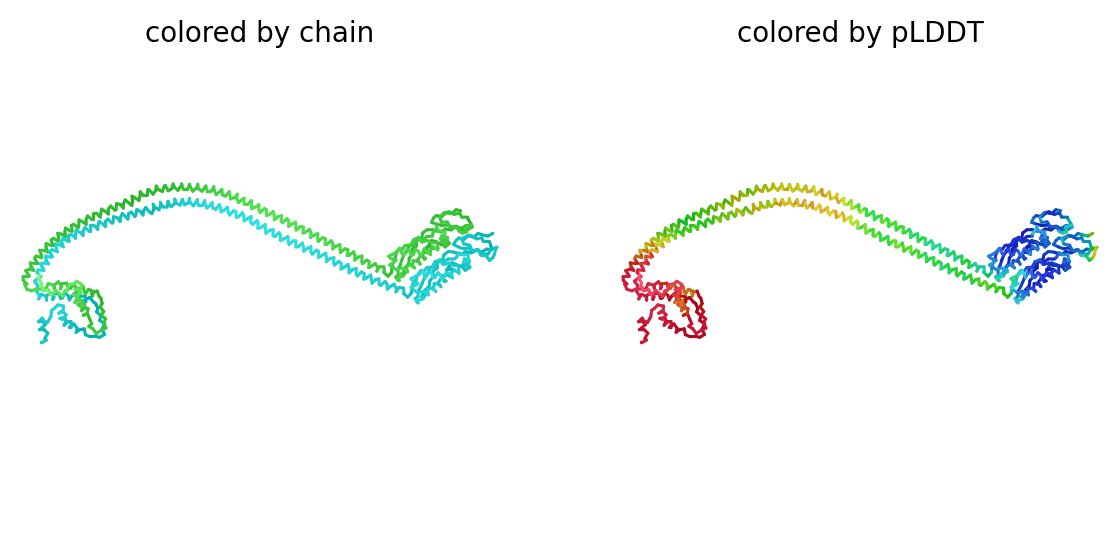

Supplement: Supplementary file 12 — Dataset 9 [file 41467_2022_33951_MOESM12_ESM.zip › FLOT1_FLOT2_dimer/rank_5_model_5_ptm_seed_0.png]
