# Supplementary material for: Cross-linking of the endolysosomal system reveals potential flotillin structures and cargo
Source: Nat Commun. 2022 Oct 20;13:6212. doi: 10.1038/s41467-022-33951-0 (PMC9584938; doi:10.1038/s41467-022-33951-0)
Supplement: Supplementary file 13 — Reporting Summary [file 41467_2022_33951_MOESM13_ESM.pdf]

nature portfolio

Corresponding author(s): Dominic Winter

Last updated by author(s): Sep 23, 2022

Reporting Summary

Nature Portfolio wishes to improve the reproducibility of the work that we publish. This form provides structure for consistency and transparency in reporting. For further information on Nature Portfolio policies, see our Editorial Policies and the Editorial Policy Checklist.

Statistics

For all statistical analyses, confirm that the following items are present in the figure legend, table legend, main text, or Methods section.

n/a	Confirmed	
☐	☒	The exact sample size (*n*) for each experimental group/condition, given as a discrete number and unit of measurement
☐	☒	A statement on whether measurements were taken from distinct samples or whether the same sample was measured repeatedly
☐	☒	The statistical test(s) used AND whether they are one- or two-sided<br>*Only common tests should be described solely by name; describe more complex techniques in the Methods section.*
☒	☐	A description of all covariates tested
☐	☒	A description of any assumptions or corrections, such as tests of normality and adjustment for multiple comparisons
☐	☒	A full description of the statistical parameters including central tendency (e.g. means) or other basic estimates (e.g. regression coefficient) AND variation (e.g. standard deviation) or associated estimates of uncertainty (e.g. confidence intervals)
☐	☒	For null hypothesis testing, the test statistic (e.g. *F*, *t*, *r*) with confidence intervals, effect sizes, degrees of freedom and *P* value noted<br>*Give P values as exact values whenever suitable.*
☒	☐	For Bayesian analysis, information on the choice of priors and Markov chain Monte Carlo settings
☐	☒	For hierarchical and complex designs, identification of the appropriate level for tests and full reporting of outcomes
☐	☒	Estimates of effect sizes (e.g. Cohen's *d*, Pearson's *r*), indicating how they were calculated

Our web collection on statistics for biologists contains articles on many of the points above.

Software and code

Policy information about availability of computer code

Data collection	Orbitrap Fusion Lumos Tune Application (3.4), Xcalibur (4.2)
Data analysis	BLAST (2.9.0), ColabFold (1.2.0), Crystallography and NMR system (CNS) (1.3), Cytoscape (3.8.0), FusionCapt advance (0.84), Ggplot2 (3.3.2), GraphPad Prism (6.01), HADDOCK (2.2), Mascot (2.5.1), MaxQuant (2.0.3.0), Openxlsx (4.1.5), PCOILS (07/2018), Proteome discoverer (2.4), PSIPRED (4.0), PyMol (2.3), R (4.0.2), RStudio (1.3.1056), SCWRL (4.0), Spectronaut (14.7.20), STRING (11.0), SWISS-MODEL (09/2021), Tidyverse (1.3.0), TopoLink (05.06.2019), UniProt (05/2019), Viridis (0.5.1), XlinkX node Proteome Discoverer (2.4), Zen (3.4), XiView (07/2019)

For manuscripts utilizing custom algorithms or software that are central to the research but not yet described in published literature, software must be made available to editors and reviewers. We strongly encourage code deposition in a community repository (e.g. GitHub). See the Nature Portfolio guidelines for submitting code & software for further information.

Data

Policy information about availability of data

All manuscripts must include a data availability statement. This statement should provide the following information, where applicable:

- Accession codes, unique identifiers, or web links for publicly available datasets
- A description of any restrictions on data availability
- For clinical datasets or third party data, please ensure that the statement adheres to our policy

All MS-data have been deposited to the ProteomeXchange Consortium via the PRIDE partner repository and are publicly available as of the date of publication

under the identifier PXD030532. The integrative cross-link models (PDBs) for PPT1 and FLOT1-FLOT2, as well as all docking input/output parameters can be accessed from the supplementary file. The remaining data are available within the article, supplementary information. Source data are provided with this paper.

Human research participants

Policy information about studies involving human research participants and Sex and Gender in Research.

Reporting on sex and gender	N/A
Population characteristics	N/A
Recruitment	N/A
Ethics oversight	N/A

Note that full information on the approval of the study protocol must also be provided in the manuscript.

Field-specific reporting

Please select the one below that is the best fit for your research. If you are not sure, read the appropriate sections before making your selection.

☒ Life sciences ☐ Behavioural & social sciences ☐ Ecological, evolutionary & environmental sciences

For a reference copy of the document with all sections, see nature.com/documents/nr-reporting-summary-flat.pdf

Life sciences study design

All studies must disclose on these points even when the disclosure is negative.

Sample size	No sample size calculation was performed. Numbers of individual replicates were chosen to allow for calculation of statistical significance using appropriate tests.
Data exclusions	No data were excluded from data analysis.
Replication	Reproducibility of experimental findings was verified by performing replicate measurements and/or by experiments with other experimental approaches.
Randomization	No randomization was performed. Individual SCX fractions of the same sample were measured together to prevent carry over between biological samples, for single shot analyses of enrichment experiments, samples were analyzed according to their complexity.
Blinding	For mass spectrometry data analysis, blinding was not possible, as sample groups have to be defined during data analysis, and necessary, as determination of significance is solely based on automated procedures. For western blotting and microscopy blinding was not possible as samples were prepared and analyzed in small batches by the same investigator.

Reporting for specific materials, systems and methods

We require information from authors about some types of materials, experimental systems and methods used in many studies. Here, indicate whether each material, system or method listed is relevant to your study. If you are not sure if a list item applies to your research, read the appropriate section before selecting a response.

Materials & experimental systems

n/a	Involved in the study	
☐	☒	Antibodies
☐	☒	Eukaryotic cell lines
☒	☐	Palaeontology and archaeology
☒	☐	Animals and other organisms
☒	☐	Clinical data
☒	☐	Dual use research of concern

Methods

n/a	Involved in the study	
☒	☐	ChIP-seq
☒	☐	Flow cytometry
☒	☐	MRI-based neuroimaging

Antibodies

Antibodies used	Goat anti LIMP2 (1:2000) R&D systems # AF1966-SP

Antibodies used	Goat anti mouse IgG HRP coupled (1:5000) Dianova # 115-035-044<br>Goat anti rabbit IgG (H+L)- Cy3 (1:400) Dianova #111-165-144<br>Goat anti rabbit IgG HRP coupled (1:5000) Dianova # 111-035-003<br>Goat anti-mouse IgG (H+L)- Alexa Fluor 488 (1:400) Thermo Fisher Scientific # A-11029<br>Mouse anti ACT2 (1:4000) Sigma-Aldrich # A5316<br>Mouse anti ATP6V1B2 (1:1000) Santa Cruz # SC166045<br>Mouse anti CANX (1:20,000) Proteintech # 66903-1-AP<br>Mouse anti FLOT1 (1:200) BD Biosciences # 610821<br>Mouse anti FLOT2 (1:1500) Proteintech # 66881-1-Ig<br>Mouse anti FLOT2 (1:200) BD Biosciences # 610383<br>Mouse anti FZD9 (1:1500) Proteintech # 67023-1-Ig<br>Mouse anti GAPDH (1:2500) Cell signaling # 5174<br>Mouse anti GM130 (1:1000) BD Biosciences # 610822<br>Mouse anti LAMP2 (1:1000) Hybridoma Bank # H4B4<br>Rabbit anti ATP6V1A1 (1:2000) Thermo Fisher Scientific # PA5-29191<br>Rabbit anti ATP6V1D (1:1000) Proteintech # 14920-1-AP<br>Rabbit anti CTSD (1:1000) Proteintech # 21327-1-AP<br>Rabbit anti DSSO (1:5000) Self-made (Singh et al., 2021-https://doi.org/10.1021/acs.analchem.0c04043)<br>Rabbit anti EEA1 (1:200) Cell signaling # 2411<br>Rabbit anti FLOT1 (1:2000) Proteintech # 15571-1-AP<br>Rabbit anti FLOT1 (1:200) Cell signaling # 18634<br>Rabbit anti GNB4 (1:2000) Proteintech # 11978-2-AP<br>Rabbit anti LAMP2 (1:400) Thermo Fisher Scientific # PA1-655<br>Rabbit anti LAMTOR1 (1:1000) Sigma-Aldrich # HPA002997<br>Rabbit anti LPHN1 (1:200) Thermo Fisher Scientific # PA5-77475<br>Rabbit anti LPHN2 (1:100) Novus biologicals #NBP2-58704<br>Rabbit anti LPHN3 (1:200) Novus biologicals #NLS1138<br>Rabbit anti RRAGA (1:1000) Cell signalling # 4357<br>Rabbit anti SDHA (1:800) Proteintech # 14865-1-AP<br>Rabbit anti TUBA (1:2000) Rockland # 600-401-880<br>Rat anti FLAG-HRP coupled (1:10,000) Sigma-Aldrich # SAB4200119
Validation	Goat anti LIMP2 (WB, ELISA), DOI: 10.3390/cancers13143520, for more visit the web page: https://www.rndsystems.com/products/human-limpii-sr-b2-antibody_af1966#product-citations<br>Mouse anti ACT2 (WB, ELISA, IF), DOI: 10.1158/0008-5472.CAN-03-4030, for more visit the web page: https://www.sigmaaldrich.com/DE/de/search/mfcd00164531<br>Mouse anti ATP6V1B2 (WB, ELISA, IF, IHC, IP), DOI: 10.1038/nchembio.2342, for more visit the web page: https://www.scbt.com/p/v-atpase-b2-antibody-d-11<br>Mouse anti CANX (WB, IF, IHC), DOI: 10.1080/09537104.2022.2108541, for more visit the web page: https://www.scbt.com/p/v-atpase-b2-antibody-d-11<br>Mouse anti FLOT1 (WB, IF), DOI: 10.1186/s12964-021-00751-w, for more visit the web page: https://www.labome.com/product/BD-Biosciences/610821.html<br>Mouse anti FLOT2 (WB, IF), DOI: 10.3892/ijo.2022.5405, for more visit the web page: https://www.ptglab.com/products/Flotillin-2-Antibody-66881-1-Ig.htm#publications<br>Mouse anti FLOT2 (WB, IF, IP), DOI: 10.1080/20013078.2017.1305677, for more visit web page: https://www.labome.com/product/BD-Biosciences/610383.html<br>Mouse anti FZD9 (WB, IHC), for more visit web page: https://www.ptglab.com/products/Frizzled-9-Antibody-67023-1-Ig.htm<br>Mouse anti GAPDH (WB, IHC, IF), DOI: 10.1152/physiolgenomics.00025.2005, for more visit web page: https://www.cellsignal.com/products/primary-antibodies/gapdh-d16h11-xp-rabbit-mab/5174<br>Mouse anti GM130 (WB, IHC, IF, IP), DOI: 10.1083/jcb.200205115, for more visit web page: https://www.bdbiosciences.com/en-de/products/reagents/microscopy-imaging-reagents/immunofluorescence-reagents/purified-mouse-anti-gm130.610822<br>Mouse anti LAMP2 (WB, IHC, IF, IP, FC), DOI: 10.1091/mbc.E17-02-0112, for more visit web page: https://dshb.biology.uiowa.edu/H4B4<br>Rabbit anti ATP6V1A1 (WB, IHC, IF, IP), DOI: 10.1042/CS20210821, for more visit web page: https://www.thermofisher.com/antibody/product/ATP6V1A-Antibody-Polyclonal/PA5-29191<br>Rabbit anti ATP6V1D (WB, IHC, IF, FC), DOI: 10.1021/pr3009385, for more visit web page: https://www.ptglab.com/products/ATP6V1D-Antibody-14920-1-AP.htm#publications<br>Rabbit anti CTSD (WB, IHC), DOI: 10.1016/j.molcel.2022.05.009, for more visit web page: https://www.ptglab.com/products/CTSD-Antibody-21327-1-AP.htm#publications<br>Rabbit anti DSSO (WB, IF), DOI: 10.1021/acs.analchem.0c04043<br>Rabbit anti EEA1 (WB, IF, IP), DOI: 10.1016/j.bbadis.2022.166496, for more visit web page: https://www.cellsignal.de/products/primary-antibodies/eea1-antibody/2411<br>Rabbit anti FLOT1 (Proteintech) (WB, IF, IP, FC), DOI: 10.1016/j.jep.2019.03.034, for more visit web page: https://www.ptglab.com/products/FLOT1-Antibody-15571-1-AP.htm#publications<br>Rabbit anti FLOT1 (Cell signaling) (WB, IHC, IF, IP), DOI: 10.1038/s41467-022-28438-x, for more visit web page: https://www.cellsignal.de/products/primary-antibodies/flotillin-1-d2v7j-xp-rabbit-mab/18634<br>Rabbit anti GNB4 (WB, IF, IP), for more visit web page: https://www.ptglab.com/products/GNB4-Antibody-11978-2-AP.htm<br>Rabbit anti LAMP2 (WB, IHC, IF), DOI: 10.1016/j.isci.2021.102949, for more visit web page: https://www.thermofisher.com/antibody/product/PA1-655<br>Rabbit anti LAMTOR1 (WB, IHC), DOI: 10.1242/jcs.100073, for more visit web page: https://www.sigmaaldrich.com/DE/de/product/sigma/hpa002997<br>Rabbit anti LPHN1 (WB, IHC, IF), for more visit web page: https://www.thermofisher.com/antibody/product/LPHN1-extracellular-Antibody-Polyclonal/PA5-77475<br>Rabbit anti LPHN2 (WB, IF), for more visit web page: https://www.novusbio.com/products/latrophilin-2-lphn2-antibody_nbp2-58704<br>Rabbit anti LPHN3 (WB, IP, FC), DOI: 10.1080/15622975.2020.1809014, for more visit web page: https://www.ptglab.com/products/LPHN3-Antibody-20045-1-AP.htm#publications

Rabbit anti RRAGA (WB, IP), DOI: 10.1016/j.jbc.2022.102030, for more visit web page: https://www.cellsignal.de/products/primary-antibodies/raga-d8b5-rabbit-mab/4357
Rabbit anti SDHA (WB, IHC, IF, IP), DOI: 10.1093/nar/gkz218, for more visit web page: https://www.ptglab.com/products/SDHA-Antibody-14865-1-AP.htm
Rabbit anti TUBA (WB, IF, ELISA), for more visit web page: https://www.citeab.com/antibodies/1906253-600-401-880-anti-alpha-tubulin-rabbit-antibody-6
Rat anti FLAG-HRP coupled (WB, IF, IP, FC), DOI: 10.1016/j.virusres.2018.10.003, for more visit web page: https://www.sigmaaldrich.com/DE/de/product/sigma/sab4200071

Eukaryotic cell lines

Policy information about cell lines and Sex and Gender in Research

Cell line source(s)	HEK293 (ATCC, CRL-1573)<br>HeLa (ATCC, CCL-2)
Authentication	None of the cell lines used were authenticated.
Mycoplasma contamination	All cell lines were tested negative for mycoplasma contamination.
Commonly misidentified lines<br>(See ICLAC register)	No commonly misidentified lines were used.
