# Supplementary material for: Cross-linking of the endolysosomal system reveals potential flotillin structures and cargo
Source: Nat Commun. 2022 Oct 20;13:6212. doi: 10.1038/s41467-022-33951-0 (PMC9584938; doi:10.1038/s41467-022-33951-0)
Supplement: Supplementary file 14 — Source Data [file 41467_2022_33951_MOESM14_ESM.zip › Source Data/Source Data File.pdf]

Full Western blots – Related to Figure 1 c

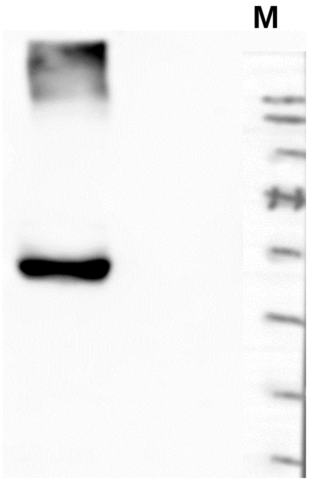

TUBA

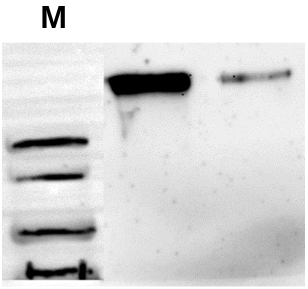

GM130

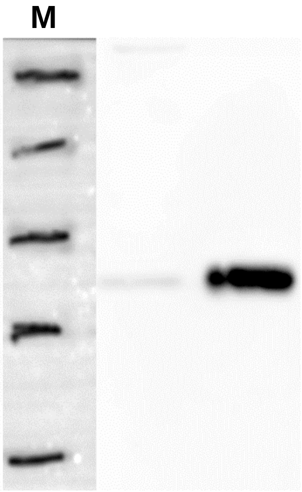

CTSD

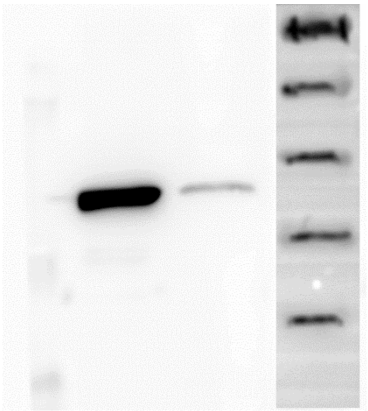

GAPDH

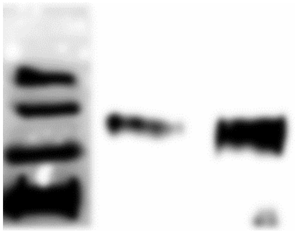

LAMP2

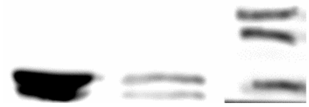

CANX

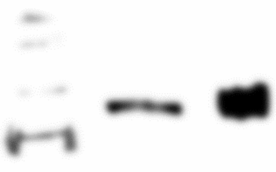

LIMP2

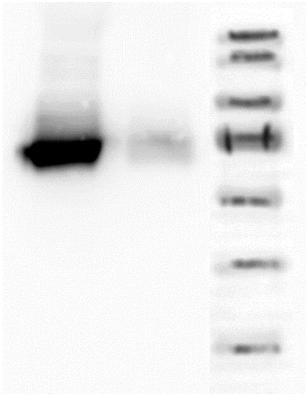

SDHA

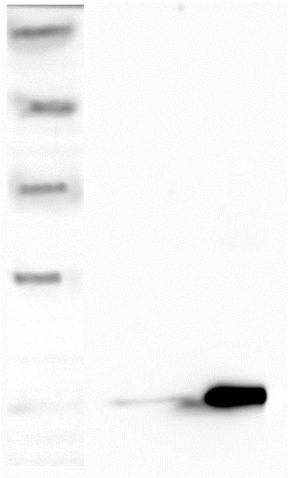

LAMTOR1

Full Silverstained SDS-gel – Related to Supplementary Figure 1 b

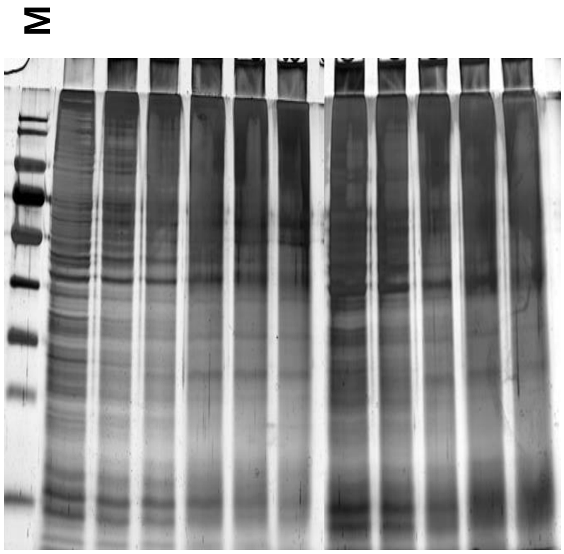

Full Western blot – Related to Supplementary Figure 1 C

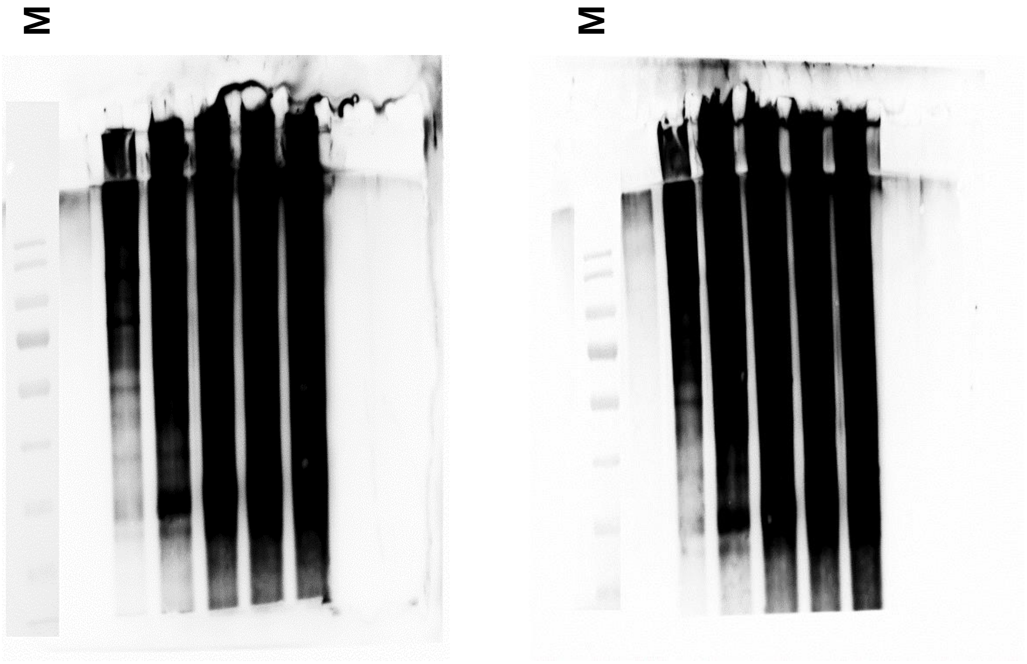

Full Western blots – Related to Figure 2 f

FZD9

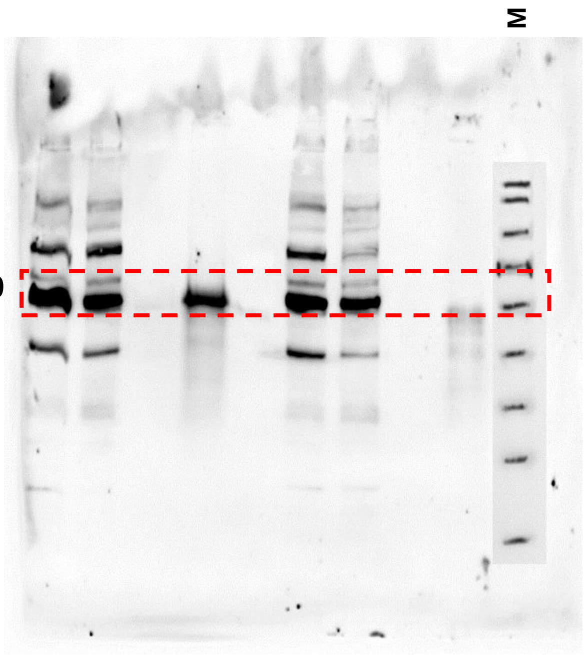

LAMP2

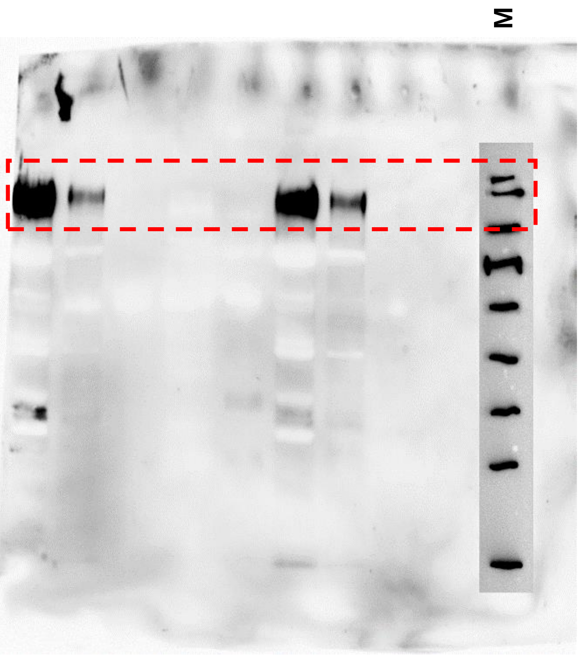

ATP6V1
B2

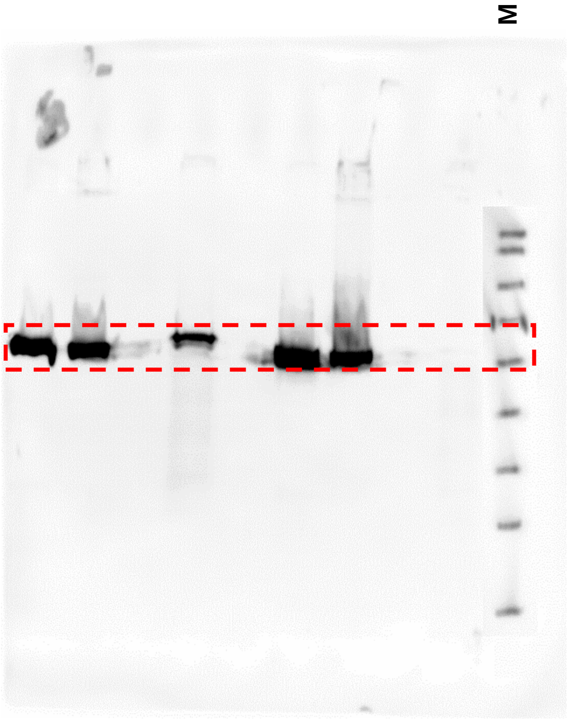

ATP6V
A1

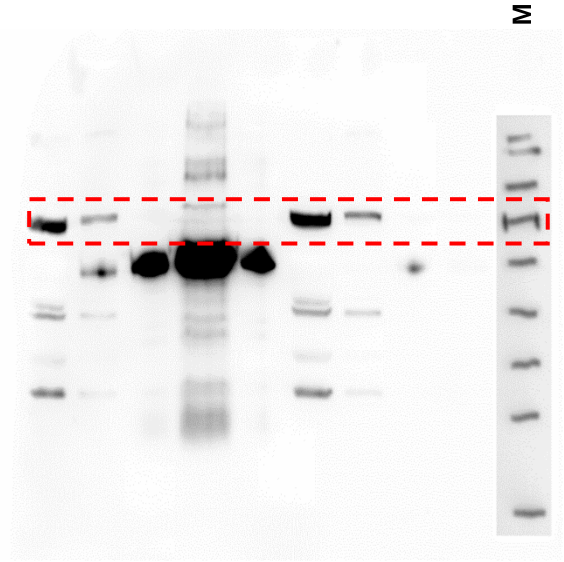

ATP6V1
A1

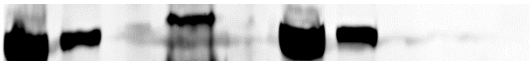

Higher exposure time (Cut)

Full Western blots – Related to Figure 2 g

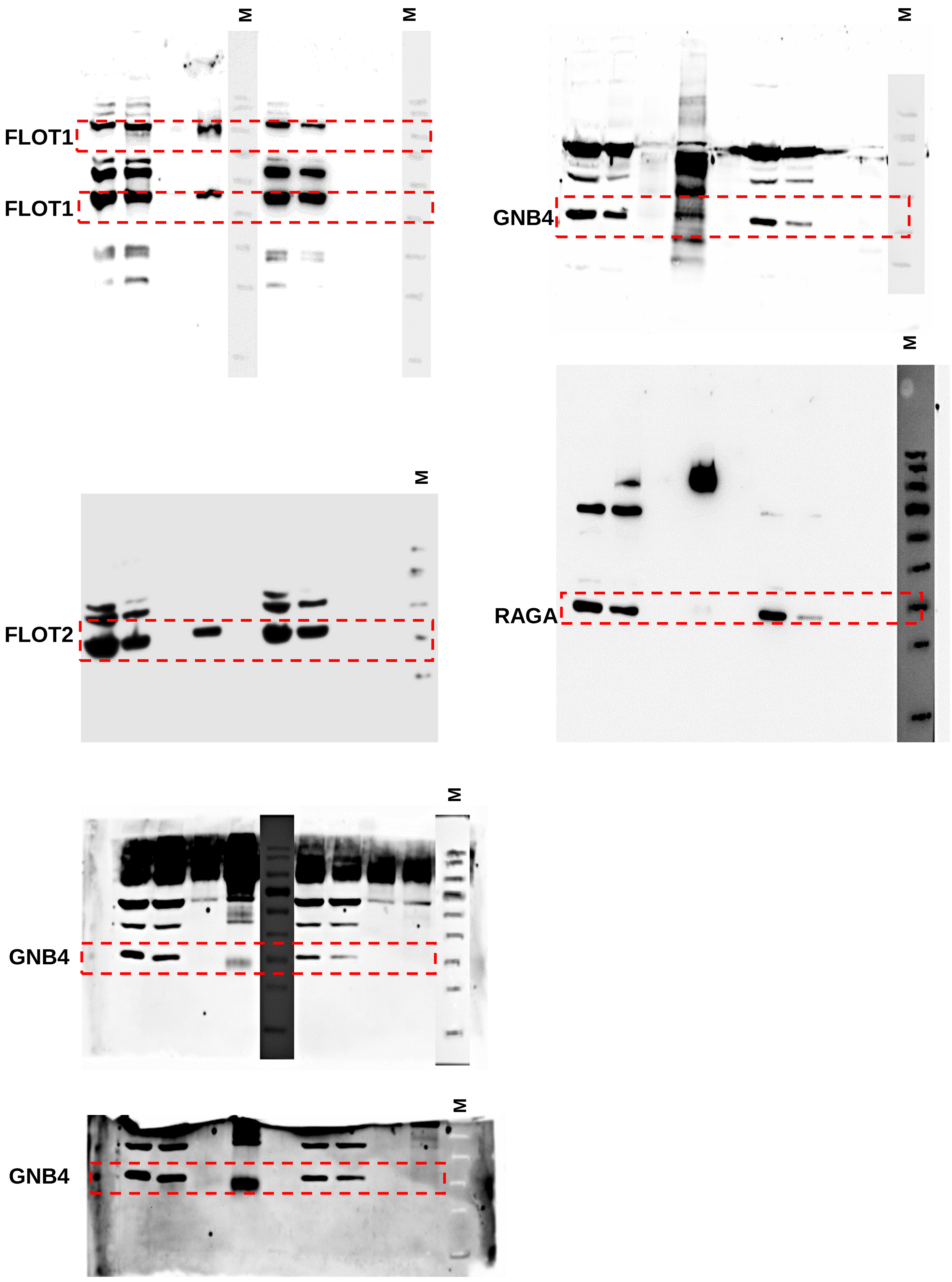

Higher exposure time (Cut)

Full Western blots – Related to Figure 4 a

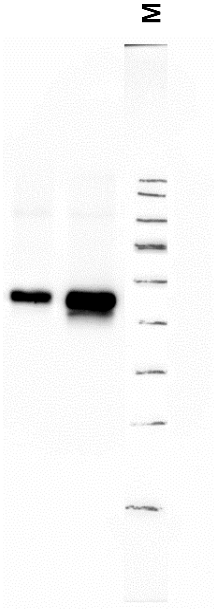

FLOT1

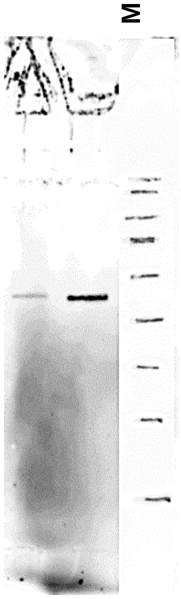

FLOT2

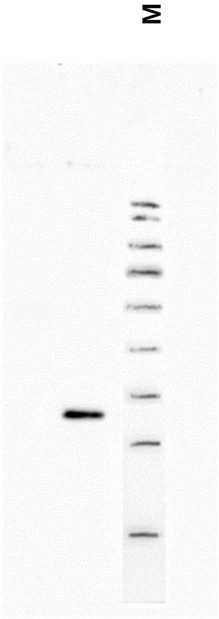

CTSD

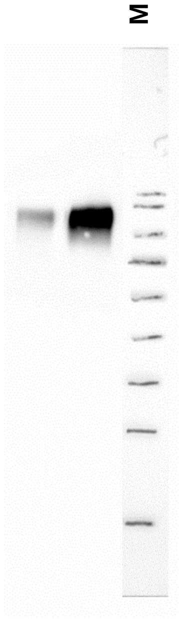

LAMP2

Full Western blots – Related to Figure 4 d

FLAG

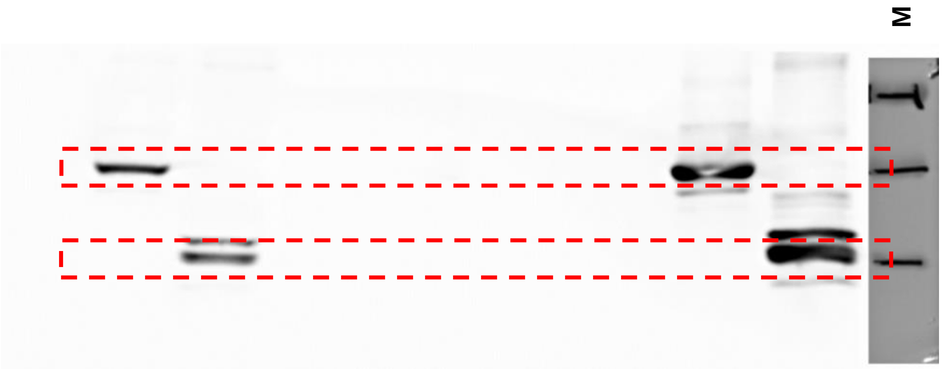

FLOT1

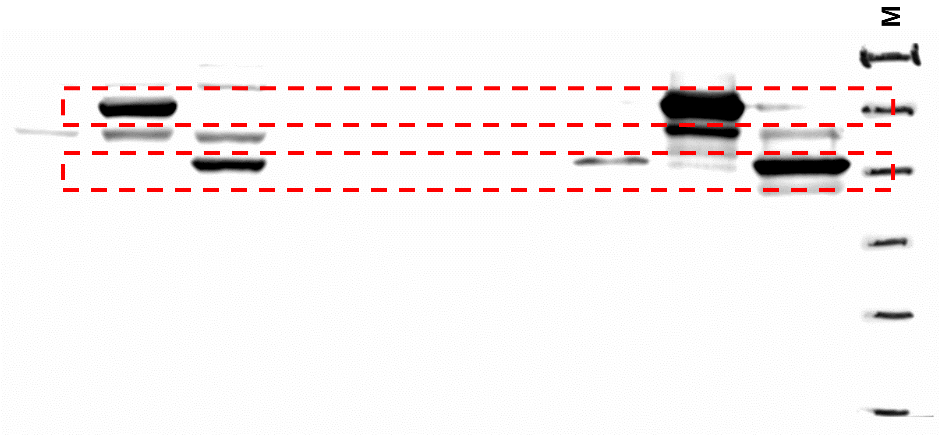

FLOT2

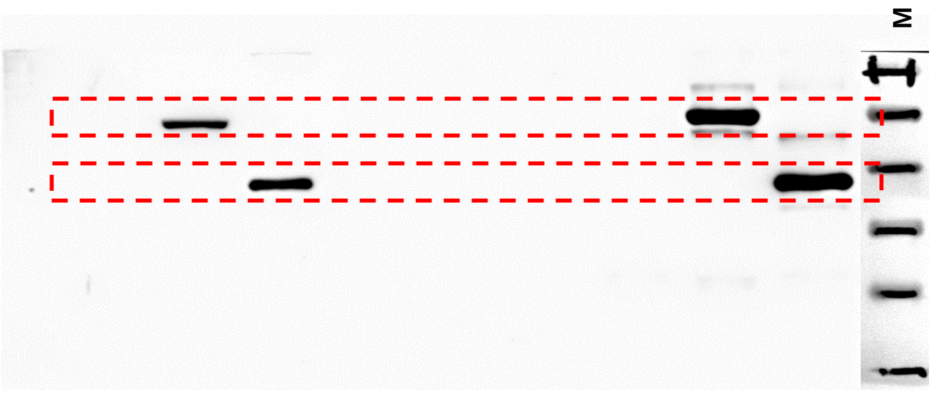

GNB4

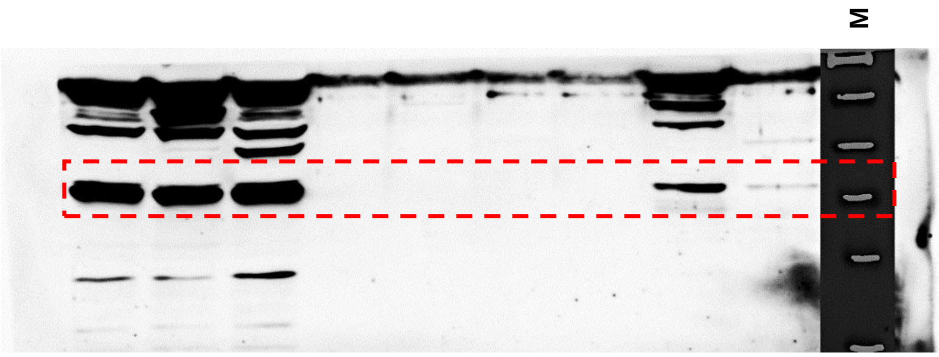

Full Western blots – Related to Figure 5 b

M

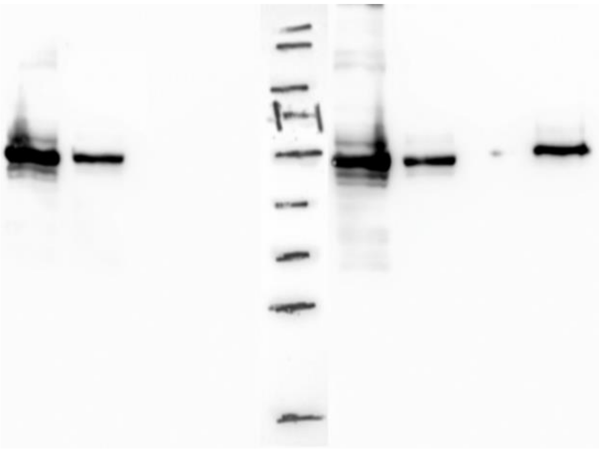

FLAG

M

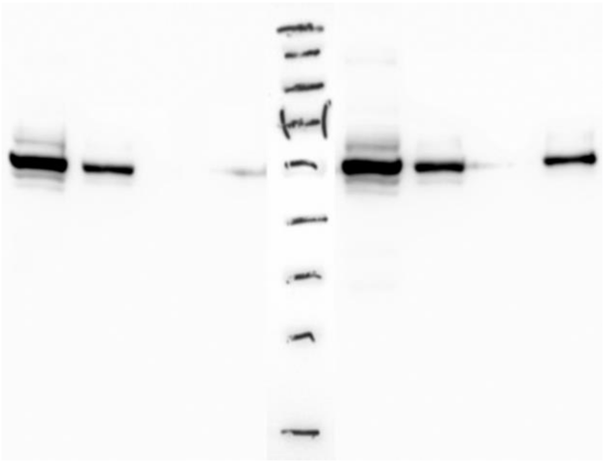

Pre-CTSD

M

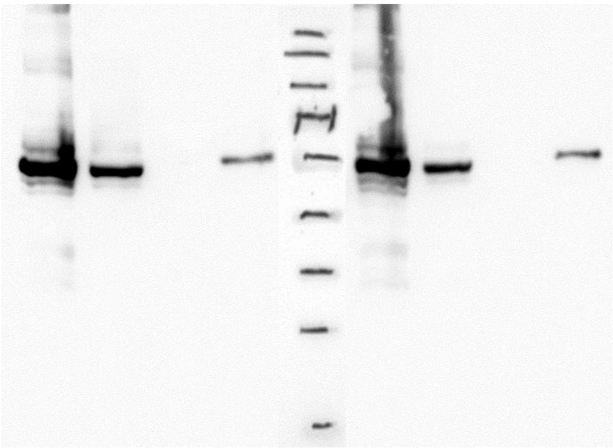

FLAG

M

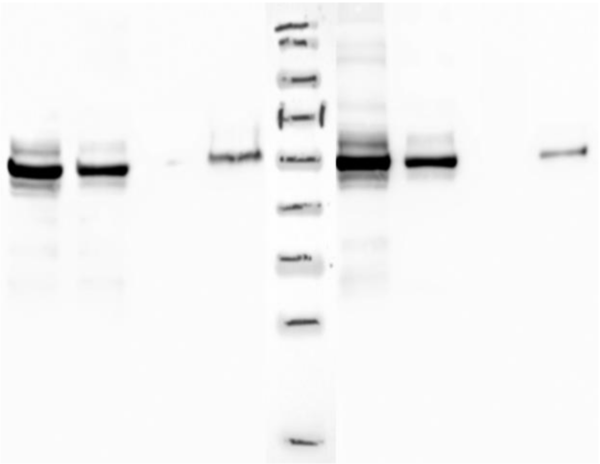

Pre-CTSD

M

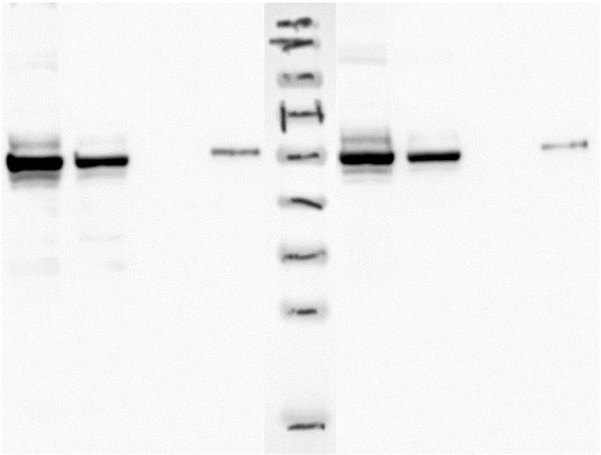

FLAG

M

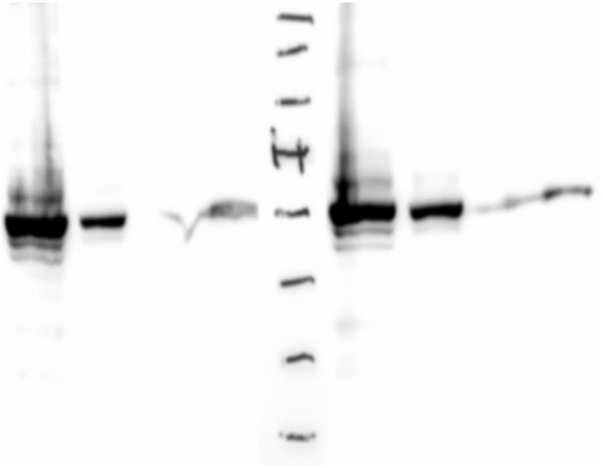

Pre-CTSD

Full Western blots – Related to Figure 5 c

M

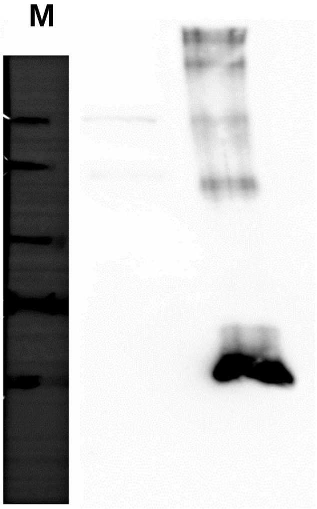

FLOT

M

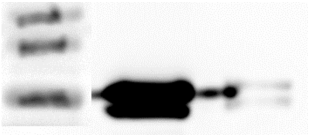

CANX

M

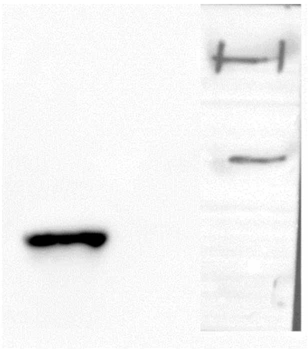

ACTG2

M

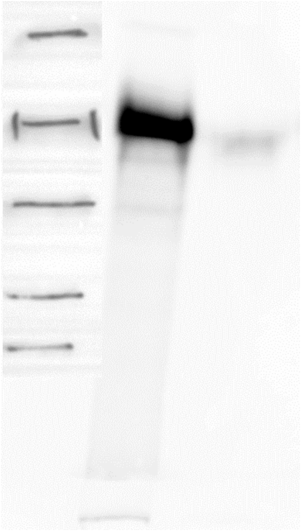

SDHA

M

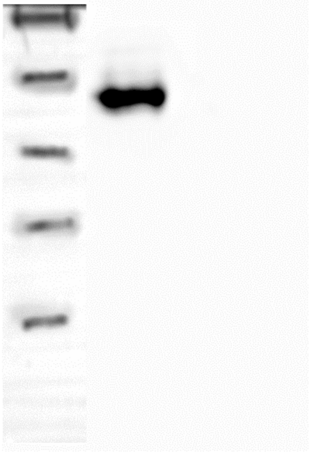

TUBA

M

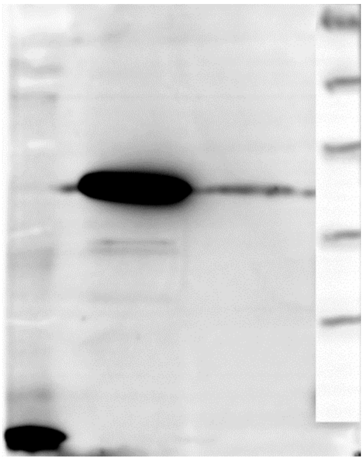

GAPDH

Full Western blots – Related to Supplementary Figure 6 b

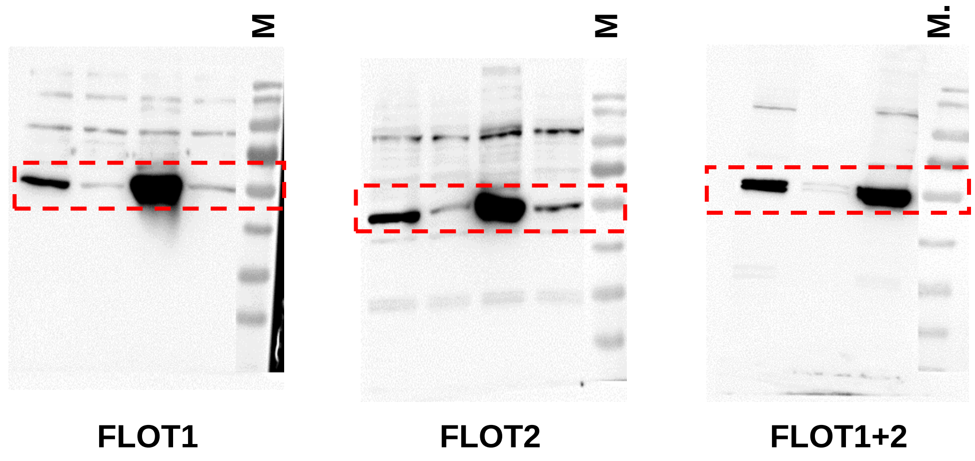

Full Western blots – Related to Supplementary Figure 6 c

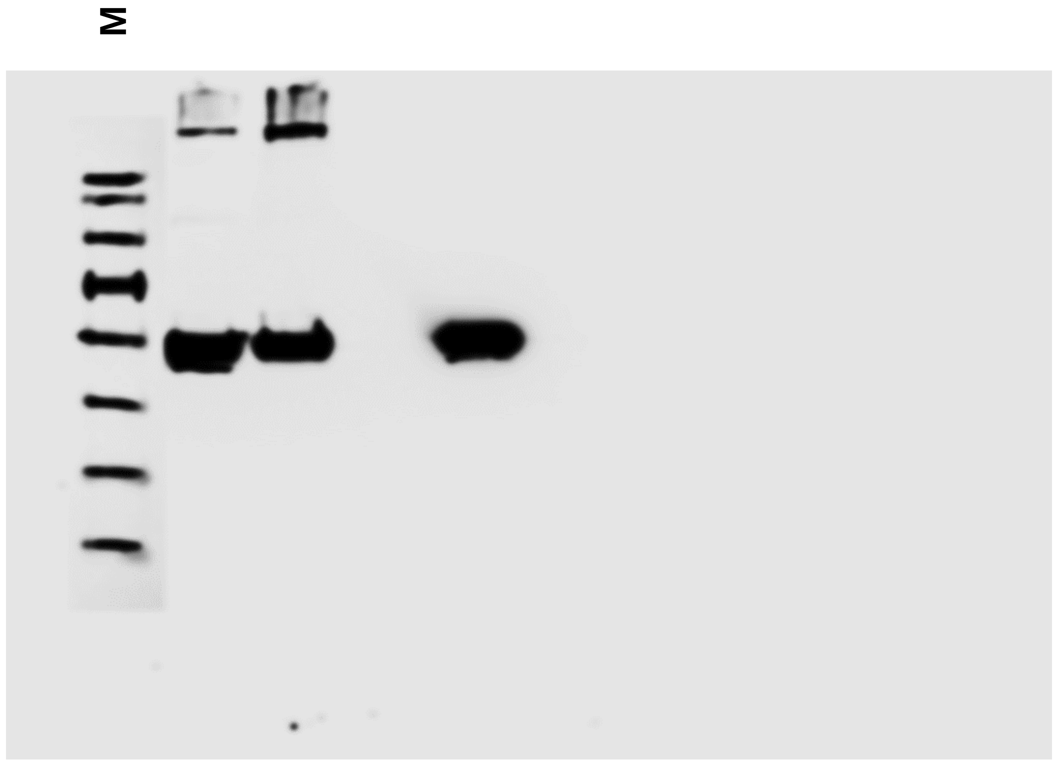

Full Western blots – Related to Figure 7 a

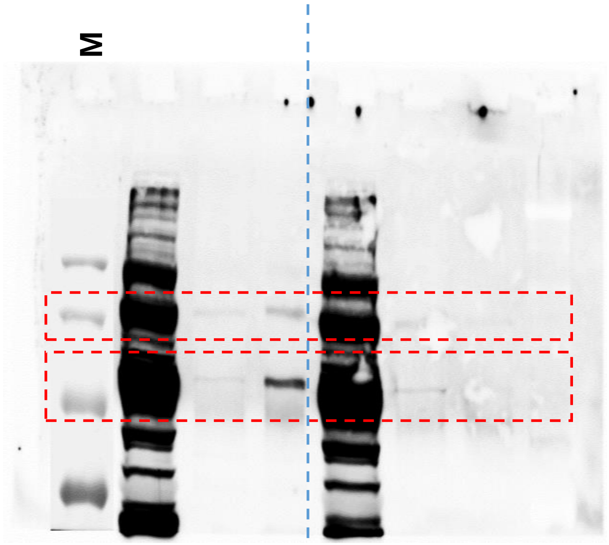

MET

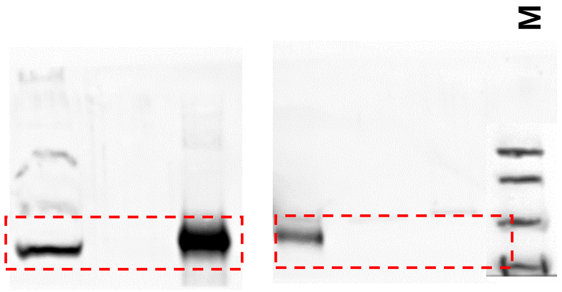

FGFR1 FGFR1

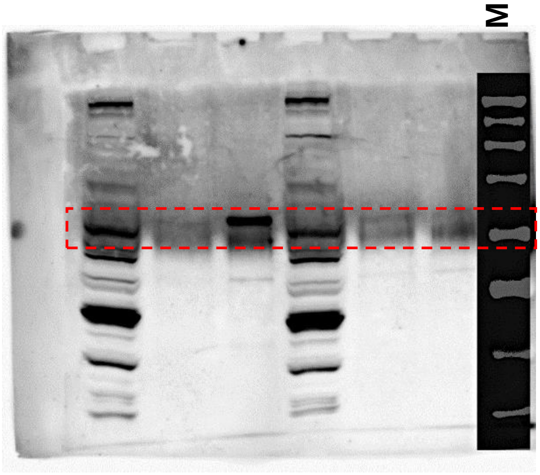

LGR4

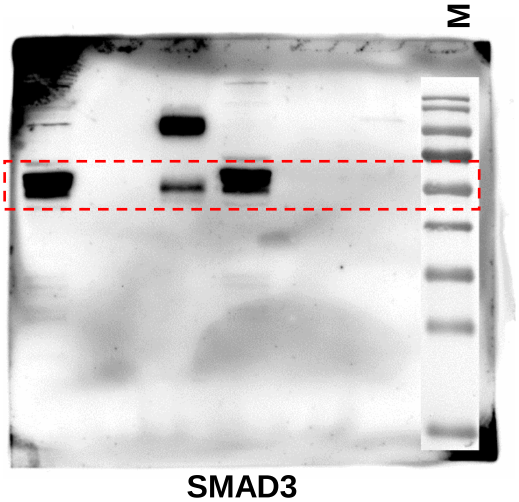

SMAD3

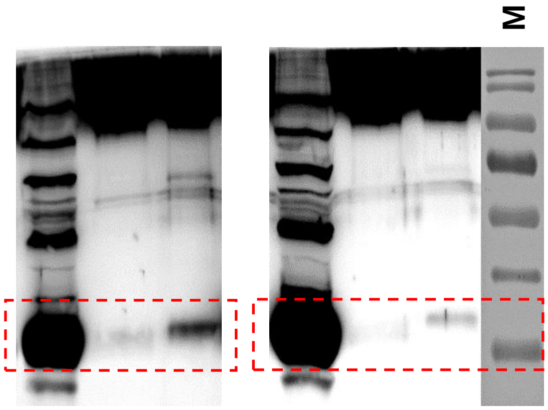

PLSCR1 PLSCR1

Full Western blots – Related to Figure 7 a

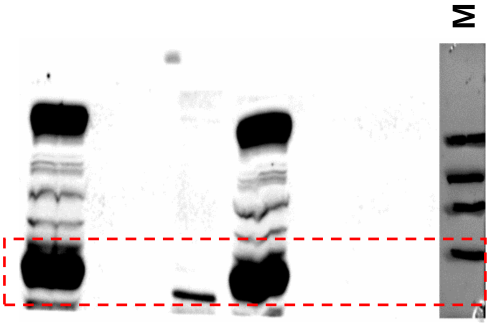

NOTCH1

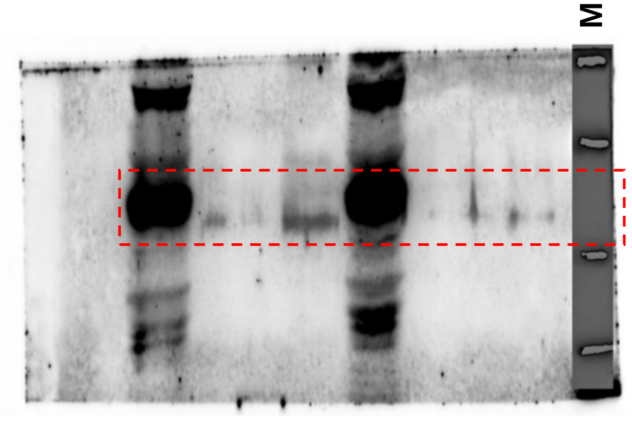

VANGL1

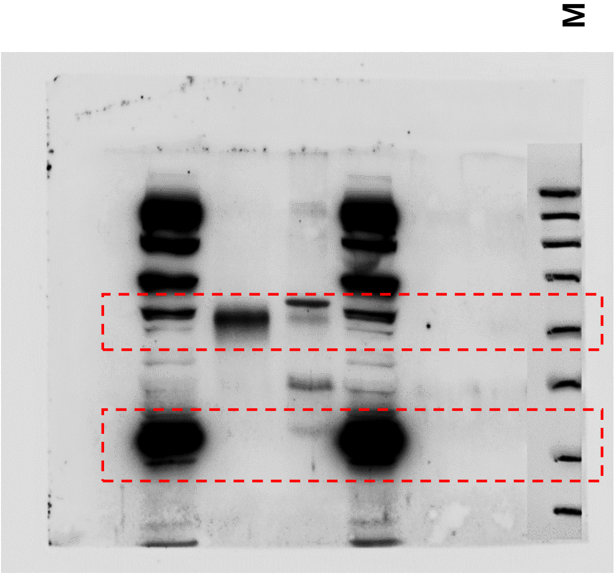

NEO1

Full Western blots – Related to Figure 7 c

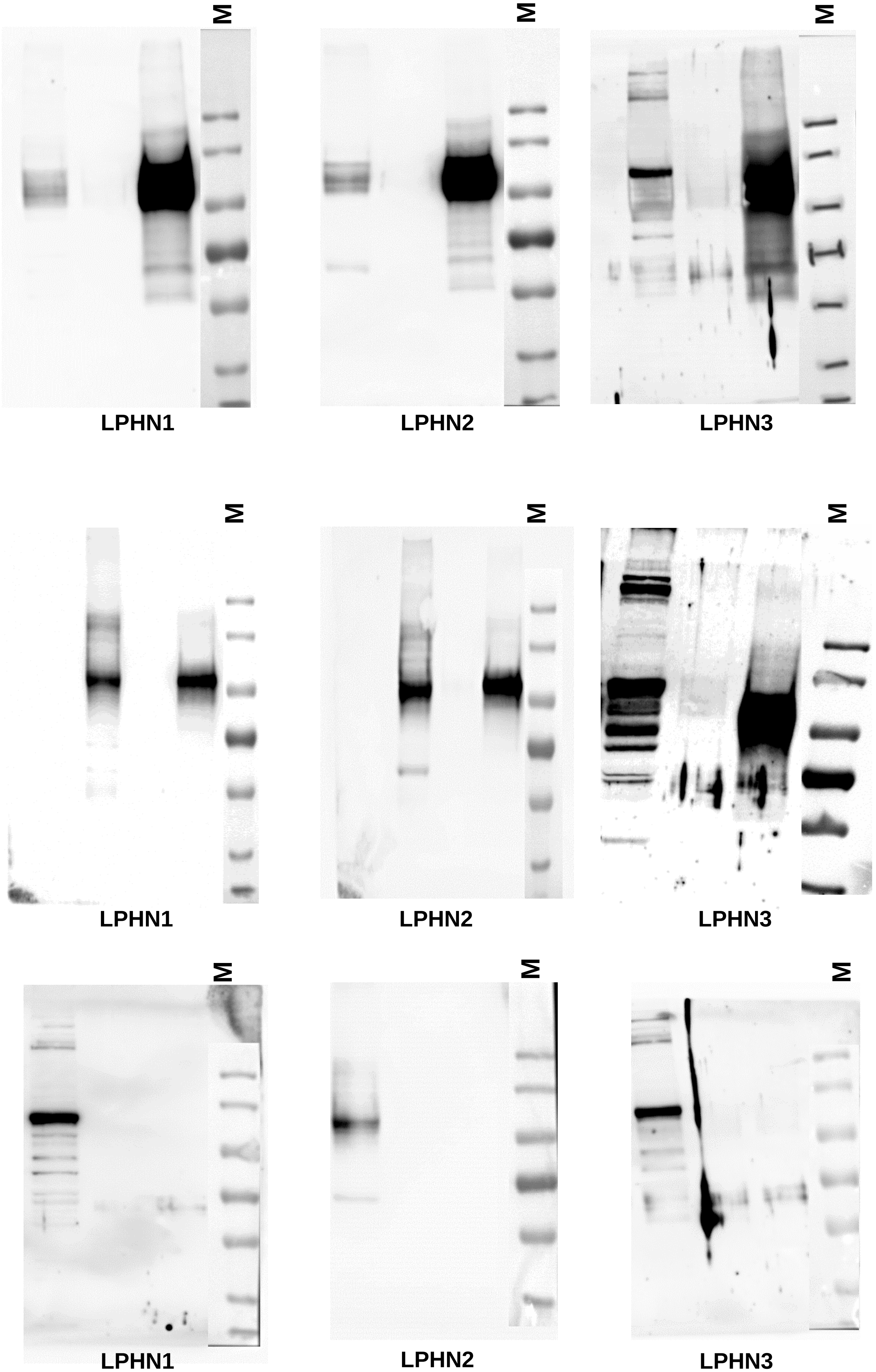

Full Western blots – Related to Figure 7 c

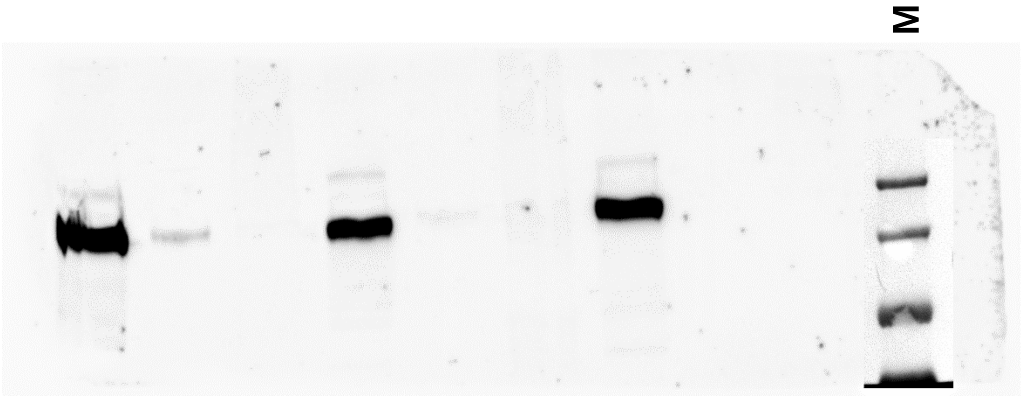

LDLR

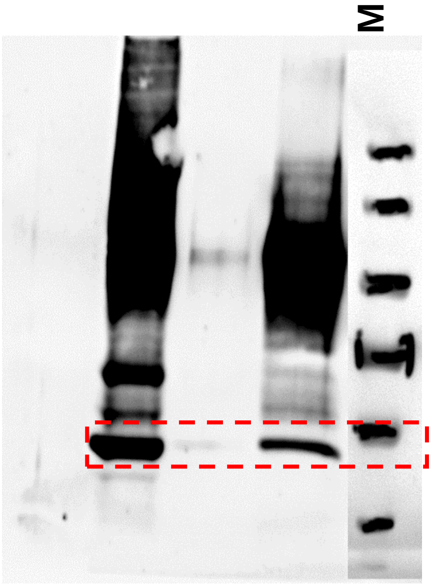

FLOT2

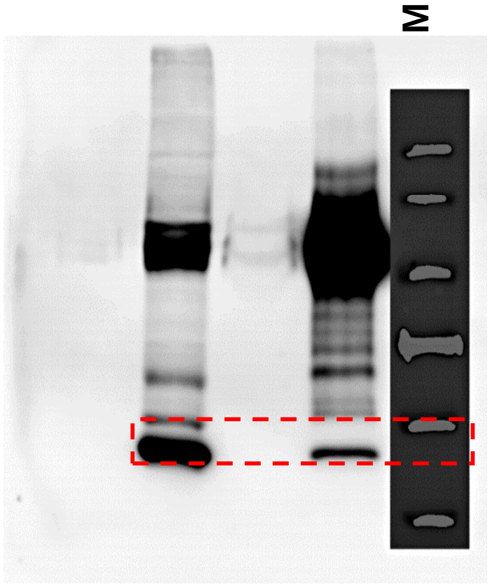

FLOT1

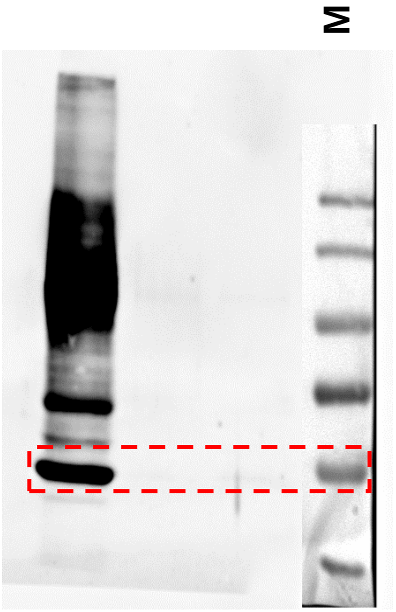

FLOT2

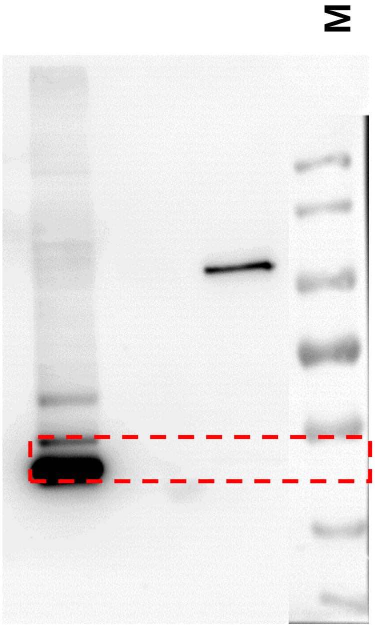

FLOT1
